# MOF Linker Extension Strategy for Enhanced Atmospheric Water Harvesting

**DOI:** 10.1021/acscentsci.3c00018

**Published:** 2023-03-06

**Authors:** Nikita Hanikel, Daria Kurandina, Saumil Chheda, Zhiling Zheng, Zichao Rong, S. Ephraim Neumann, Joachim Sauer, J. Ilja Siepmann, Laura Gagliardi, Omar M. Yaghi

**Affiliations:** † Department of Chemistry, University of California, Berkeley, California 94720, United States; ‡ Kavli Energy Nanoscience Institute, University of California, Berkeley, California 94720, United States; § Bakar Institute of Digital Materials for the Planet, Division of Computing, Data Science, and Society, University of California, Berkeley, California 94720, United States; ⊥ Institut für Chemie, Humboldt-Universität zu Berlin, Berlin 10099, Germany; ¶ Department of Chemical Engineering and Materials Science, Department of Chemistry, and Chemical Theory Center, University of Minnesota—Twin Cities, Minneapolis, Minnesota 55455, United States; □ Department of Chemistry, Pritzker School of Molecular Engineering, and Chicago Center for Theoretical Chemistry, University of Chicago, Chicago, Illinois 60637, United States; ∥ KACST−UC Berkeley Center of Excellence for Nanomaterials for Clean Energy Applications, King Abdulaziz City for Science and Technology, Riyadh 11442, Saudi Arabia

## Abstract

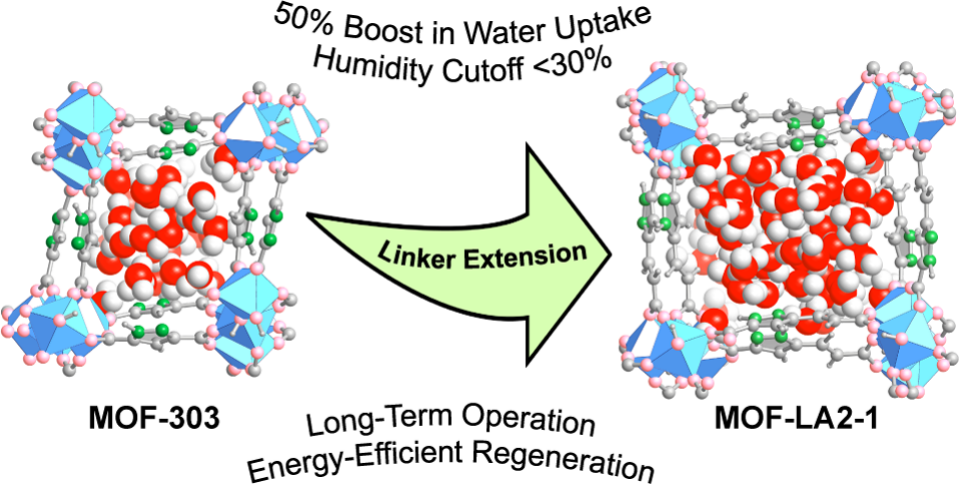

A linker extension strategy for generating metal–organic frameworks (MOFs) with superior moisture-capturing properties is presented. Applying this design approach involving experiment and computation results in MOF-LA2-1 {[Al(OH)(PZVDC)], where PZVDC^2–^ is (*E*)-5-(2-carboxylatovinyl)-1*H*-pyrazole-3-carboxylate}, which exhibits an approximately 50% water capacity increase compared to the state-of-the-art water-harvesting material MOF-303. The power of this approach is the increase in pore volume while retaining the ability of the MOF to harvest water in arid environments under long-term uptake and release cycling, as well as affording a reduction in regeneration heat and temperature. Density functional theory calculations and Monte Carlo simulations give detailed insight pertaining to framework structure, water interactions within its pores, and the resulting water sorption isotherm.

## Abstract 2

A linker extension strategy leading to a MOF with 50% increased water uptake capacity at low relative humidity, good long-term hydrothermal stability, and energy-efficient regeneration is presented.

## Introduction

Water stress affects about half of the world population.^1,2^ Given the presence of clean water in the atmosphere, porous and hygroscopic sorbents are being investigated for water extraction from air.^3,4^ An ideal water-harvesting material should (i) take up water at a desirable relative humidity (RH), including from desert air, (ii) exhibit step-shaped moisture uptake behavior to allow for uptake and release of large amounts of water by minor perturbations in temperature or pressure, (iii) display facile water release to reduce the energy consumption and increase the productivity, (iv) have hydrothermal stability to enable long-term operation, and (v) be made from nontoxic, abundant components using environmentally benign processes.

**Figure 1:**
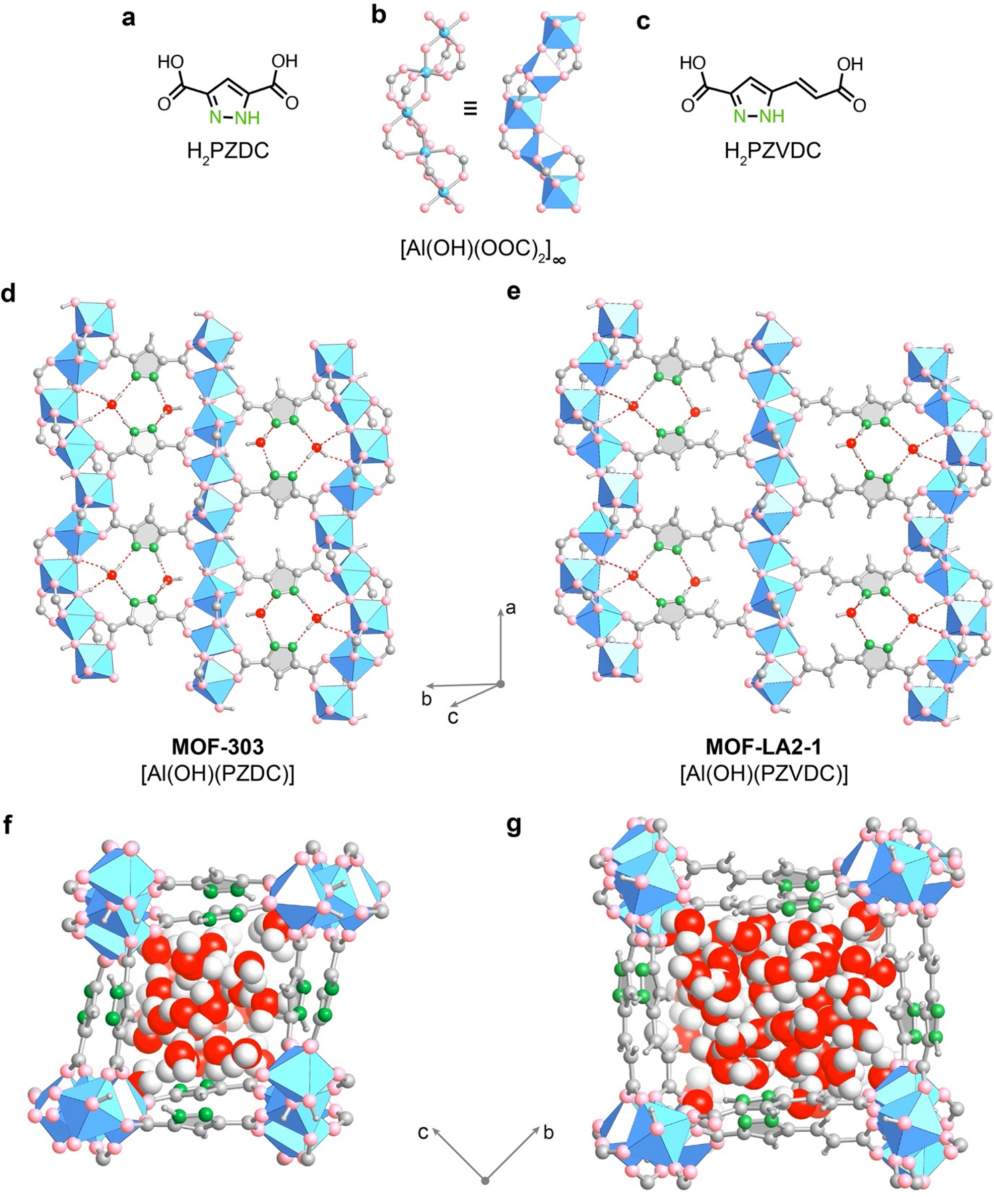
Comparison of the framework structures and water arrangements in MOF-303 (left) and MOF-LA2-1 (right). (a) The linker 1*H*-pyrazole-3,5-dicarboxylic acid (H_2_PZDC) of MOF-303. (b) The aluminum oxide SBU of both MOFs consists of alternating *cis*–*trans*-corner-shared AlO_6_ octahedra. (c) The linker (*E*)-5-(2-carboxyvinyl)-1*H*-pyrazole-3-carboxylic acid (H_2_PZVDC) of MOF-LA2-1, where LA2-1 refers to long-arm extension of the linker by two carbon atoms on one side. (d, e) A cut-away view of the pores displaying the alignment of the pyrazole-based linkers such that their hydrophilic N(H) functionalities point toward each other, thus generating an alternating pattern of hydrophilic and hydrophobic pockets. The framework structures and water positions were obtained by a combination of X-ray diffraction analysis and DFT optimization. The hydrophilic pockets serve as strong adsorption sites, which are displayed at a loading of two water molecules per respective asymmetric unit [Al(OH)(PZDC)]_2_ (d) and [Al(OH)(PZVDC)]_2_ (e). (f, g) Snapshots of the water structures from Monte Carlo simulations at saturated water loadings of 10 and 18 molecules per asymmetric unit in MOF-303 (f) and MOF-LA2-1 (g), respectively, displayed along the pore channel. Coordinate systems are given for guidance. Al, blue octahedron; C and H, gray; N, green; O in framework, pink; O in H_2_O, red.

In this regard, metal–organic frameworks (MOFs) are promising materials because of the facility with which they can be designed and modified to achieve a desired property,^5−7^ which has led to their successful implementation for atmospheric water harvesting.^8−14^ In particular, the discovery of MOF-303 {[Al(OH)(PZDC)], where PZDC^2–^ is 1*H*-pyrazole-3,5-dicarboxylate; Figure 1a} represents an important advance toward meeting the above-described sorbent requirements.^11^ Specifically, the aluminum oxide rodlike secondary building units (SBUs; Figure 1b) impart hydrothermal stability to the framework and, jointly with the aligned PZDC^2–^ linkers, generate pores lined by alternating hydrophilic–hydrophobic pockets. Single-crystal X-ray diffraction analysis and *ab initio* molecular dynamics simulations revealed how these pockets are ideally suited for binding of initial water molecules that seed the evolution of the overall water structure.^15^

The conundrum solved by the present study is how to retain the alternating hydrophilic–hydrophobic pocket environment while simultaneously increasing the water uptake capacity of the framework, in other words, how to increase the pore volume of MOF-303 without compromising its favorable water-uptake attributes. The usual strategy to increase the pore volume of aluminum MOFs made from rodlike SBUs is linker extension, involving either polycyclic aromatic linkers or appending additional aromatic rings to the linker.^16−19^ However, these approaches generated either hydrophobic, less porous, or large-pore hydrolytically labile aluminum frameworks.^16,19,20^

Herein, through an integrated experimental–computational approach, we identified and implemented a suitable linker extension strategy involving appending a single vinyl group to the PZDC^2–^ linker (Figure 1a). The corresponding MOF, termed MOF-LA2-1 {[Al(OH)(PZVDC)], where PZVDC^2–^ is (*E*)-5-(2-carboxylatovinyl)-1*H*-pyrazole-3-carboxylate; Figure 1c}, is isostructural to MOF-303 featuring an increased pore volume and hence water uptake. Although MOF-LA2-1 exhibits an isothermal step shifted to higher RH compared to MOF-303, it can still be suitable for arid environments. Additionally, this MOF offers a significantly reduced regeneration temperature and enthalpy, as well as high stability upon water adsorption–desorption cycling.

## Results and Discussion

At the outset of this study, we hypothesized that addition of a relatively compact, yet long group to the hydrophilic H_2_PZDC linker utilized in MOF-303 will enhance its water uptake capacity while leveraging its hydrophilic nature and its excellent hydrothermal stability (Figure 1c). In particular, we were keen to retain the arrangement of the pyrazole functionalities, which served as primary adsorption sites and were key for its favorable water-harvesting properties (Figure 1d).^15^ Density functional theory (DFT) calculations on periodic structures consistent with the MOF-303 topology indicated that a vinyl-appended variant would offer a favorable increase in pore volume. The water adsorption isotherm simulated for the corresponding structure using Gibbs ensemble Monte Carlo (GEMC) simulations demonstrated a substantial increase in water uptake capacity with the isotherm step position suitable for water harvesting at arid conditions (Supporting Information, Section S3.1). Accordingly, the linker H_2_PZVDC featuring a vinyl group extension of H_2_PZDC was synthesized via a two-step procedure employing a Wittig reaction on ethyl 5-formyl-1*H*-pyrazole-3-carboxylate followed by hydrolysis (Supporting Information, Section S2). MOF-LA2-1 was then obtained using AlCl_3_·6H_2_O and H_2_PZVDC by employing solvothermal synthesis in a DMF/H_2_O (1:4) mixture at 120 °C and also by using a green synthesis procedure in H_2_O under reflux and stirring (Supporting Information, Section S2).

**Figure 2:**
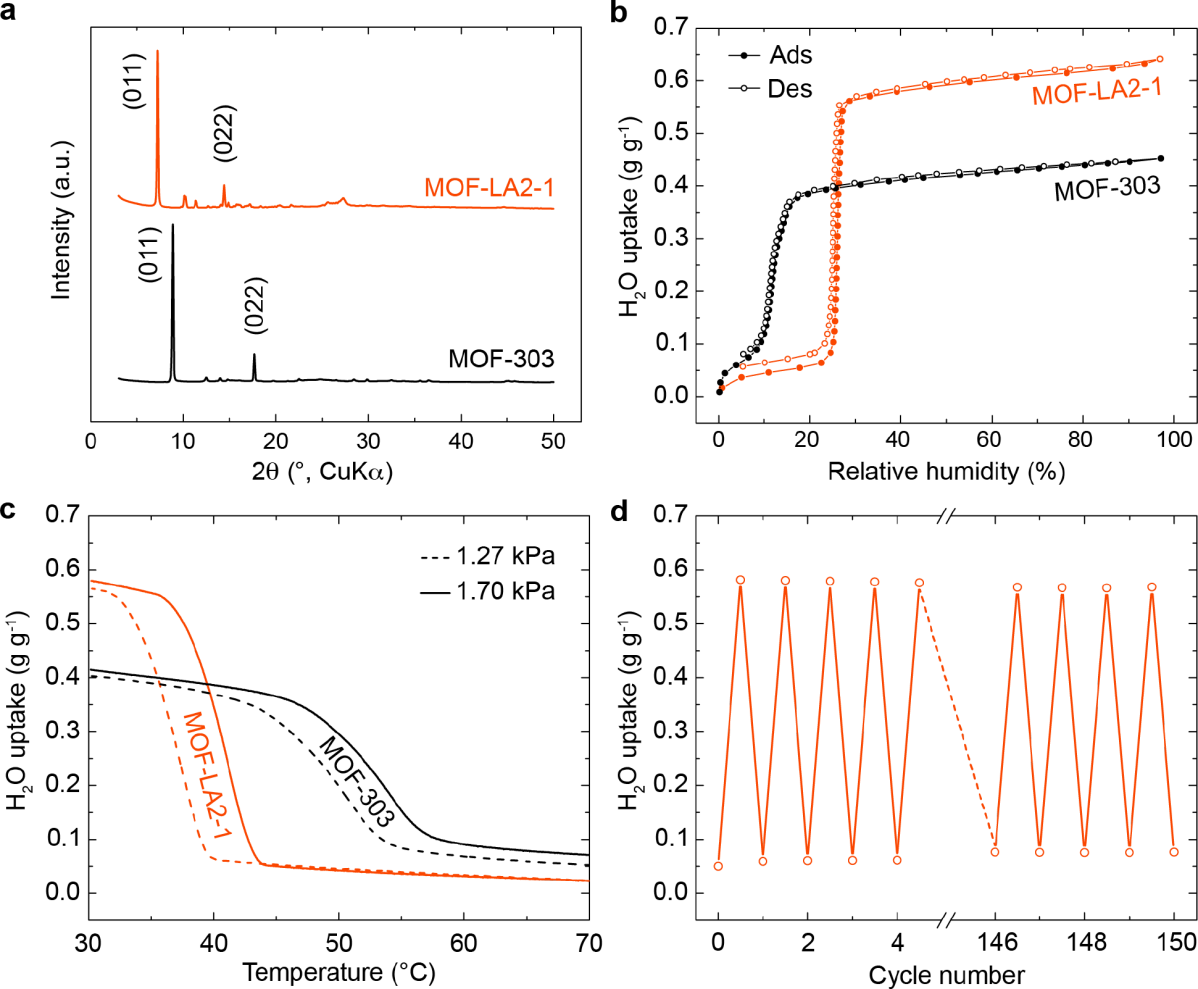
Experimental structural and water sorption analysis of MOF-LA2-1 in comparison to MOF-303. (a) Powder X-ray diffraction analysis using CuKα radiation. Major peaks are labeled according to the associated crystallographic lattice planes. (b) Water sorption isotherms at 25 °C. (c) Water desorption isobars at water vapor pressures of 1.27 and 1.70 kPa. The materials were loaded at 30 °C and at the respective water vapor pressure prior to the measurement. (d) Adsorption–desorption cycling at 1.70 kPa for 150 cycles under temperature swing between 30 and 45 °C.

The resulting microcrystalline powder was first characterized by powder X-ray diffraction (PXRD) analysis. A significant 2θ shift of the corresponding PXRD reflections to lower values compared to MOF-303 was indicative of successful isoreticular extension of the parent framework (Figure 2a). Additionally, scanning electron microscopy coupled with energy dispersive X-ray spectroscopy confirmed phase purity of the prepared sample (Supporting Information, Section S4). Significant efforts to obtain single crystals suitable for single-crystal X-ray diffraction (SCXRD) analysis of MOF-LA2-1 resulted in a crystal size of approximately 10 × 10 × 30 μm^3^ (Supporting Information, Section S5). While synchrotron SCXRD data gave us insight regarding the unit cell parameters {*a* = 12.030(12) Å, *b* = 17.398(17) Å, *c* = 17.706(17) Å, and β = 99.33(2)°} and SBU stereochemistry, we hypothesize that due to the substantial intrinsic positional disorder of the asymmetric linker in the crystal structure, the crystallinity of these crystals was relatively low, thus limiting the overall SCXRD data quality and preventing us from obtaining the exact linker configuration in MOF-LA2-1.

Thus, we utilized periodic DFT optimizations to probe the relative stability of the different possible linker configurations in the MOF-LA2-1 structure at the unit cell parameters extracted from SCXRD data (Supporting Information, Section S3.2). In this context, a total of 16 possible backbone configurations of the framework featuring different positions and orientations of the pyrazole and vinyl groups in the hydrophilic cavity of the MOF were considered. Generally, the configurations where the pyrazole functionalities were on the same side of the pocket (ZUS, from German “*zusammen*”, together; as in Figure 1e) were estimated to be more stable than the configurations with the pyrazole moieties on opposite sides of the pocket (ENT, from German “*entgegen*”, opposite). The pyrazole functionalities in the ZUS configuration of MOF-LA2-1 hydrogen bond to each other, thus stabilizing the associated structural arrangement. This is further supported by the fact that the pyrazole moieties lie in the same plane in this configuration, whereas they do not lie in a common plane in the ENT configuration. Overall, one ZUS structure (Figure 1e) was identified as particularly stable, with the next most stable configuration lying 27 kJ mol^–1^ {per asymmetric unit [Al(OH)(PZVDC)]_2_} higher in energy (Supporting Information, Section S3.2), thus likely being the most prevalent configuration of MOF-LA2-1.

As discussed earlier, MOF-LA2-1 was derived from MOF-303 by adding a vinyl group to the H_2_PZDC linker molecule with the goal of enhancing its water uptake capacity while retaining the arrangement of the pyrazole functionalities, which were determined to be key to the water-harvesting properties of MOF-303.^15^ Having determined the most stable framework configuration, we computationally investigated the primary water adsorption sites of MOF-LA2-1 in this arrangement and compared them to the respective sites in MOF-303 (Figure 1d, e). Indeed, similar to the primary water adsorption sites in MOF-303, water molecules are adsorbed in sites constituted by the linkers’ pyrazole groups as well as μ_2_-OH groups of the aluminum SBU. The first water molecule adsorbs through the formation of four hydrogen bonds (2.7–3.0 Å) with the framework—one each with the N and NH groups of the linkers and two with the μ_2_-OH groups of the aluminum SBU. The second water molecule adsorbs through two hydrogen bonds (both at 2.7 Å), each with the remaining N and NH groups (Figure 1e). These water adsorption sites in MOF-LA2-1 are similar to those observed in MOF-303 (Figure 1d). While the first water molecule binds with comparable strength, the second water molecule binds less strongly relative to MOF-303 (Supporting Information, Section S3.3), which likely contributes to the shift in the isotherm toward higher RH compared to MOF-303 (see below). The subsequent water molecule is anticipated to adsorb on the remaining μ_2_-OH group of the aluminum SBU and additional water molecules to fill the pore by forming a hydrogen-bonded network, as observed previously in MOF-303 (Figure 1f, g).^15^

Considering the insights gained through DFT calculations, we refined the structural model of MOF-LA2-1 in its most stable configuration (Figure 1e) against the experimental PXRD data (Supporting Information, Section S6). The framework was modeled in the *P*2_1_/*c* space group (No. 14), and the final unit cell parameters were refined to *a* = 12.1 Å, *b* = 17.3 Å, *c* = 17.8 Å, and β = 98.6°, with good agreement with the SCXRD data.

Next, the thermal stability and porosity of MOF-LA2-1 were studied using thermogravimetric analysis (TGA) and nitrogen sorption analysis, respectively. TGA under both argon and air atmosphere revealed no significant weight loss below 300 °C (Supporting Information, Section S7). This indicates an excellent stability required for thermal regeneration of this material. Evaluation of the nitrogen sorption isotherm at 77 K of MOF-LA2-1 yields a Brunauer–Emmett–Teller surface area and a pore volume of 1892 m^2^ g^–1^ and 0.67 cm^3^ g^–1^, respectively—values approximately 1.4 times higher compared to those of MOF-303 (Supporting Information, Section S8).^15^

The water-harvesting properties of MOF-LA2-1 were first probed by performing water sorption measurements under isothermal conditions. Similar to the parent framework, the extended framework displayed a pre-step in its isotherm, which is very likely associated with the presence of a hydrophilic pocket generated by the pyrazole functionalities, thus forming strong water adsorption sites, as was previously observed for MOF-303.^15^ Notably, the water sorption isotherm profile exhibited a steep step at 26% RH with a total water uptake of 0.64 and 0.68 g g^–1^ for the solvothermal and reflux-based synthesis method, respectively—an approximately 50% higher water capacity than that of MOF-303 (Figures 2b and S19). Although shifted to higher RH values in comparison with MOF-303, the step position of MOF-LA2-1 can still be suitable for water harvesting in most arid regions of the world.^21,22^ For conditions with RH values below the isotherm inflection point, we can envision utilization of pressurized swing adsorption or introduction of more hydrophilic groups into the linker molecule to allow for water harvesting in these hyperarid climates using MOF-LA2-1 or derivatives thereof.

In addition, we conducted water sorption analysis at different temperatures and utilized these data to assess the isosteric heat of water adsorption *Q*_st_ using the Clausius–Clapeyron relation (Supporting Information, Section S9). We found that MOF-LA2-1 exhibits an average *Q*_st_ value of 51 kJ mol^–1^—an overall reduction of 3 kJ mol^–1^ compared to its parent framework evaluated at similar conditions.^23^ Considering the heat of condensation of water (44 kJ mol^–1^ at 25 °C), this resembles a 30% lower heat of adsorption penalty compared to MOF-303. Importantly, we note that the favorable water sorption properties of MOF-LA2-1 were not compromised when using the green reflux-based synthesis for its preparation (Supporting Information, Section S9).

Furthermore, the regeneration temperature of MOF-LA2-1 was probed by measuring isobaric desorption curves. These measurements were conducted at water vapor pressures of 1.27 and 1.70 kPa (corresponding to 30 and 40% RH at 30 °C, respectively) and demonstrated substantially reduced water release temperatures compared to MOF-303 (Figure 2c), thus allowing for a very desirable operational desorption temperature of 45 °C. Together with the significantly reduced isosteric heat of adsorption, these findings substantiate MOF-LA2-1 as an energy-efficient water-harvesting material for arid regions.

To examine the stability of MOF-LA2-1 at the operational conditions, temperature swing adsorption–desorption cycling was performed at 1.70 kPa water vapor pressure (Figure 2d). This experiment showed a 5% decrease in water uptake working capacity after 75 cycles and a further 1% decrease after 75 additional cycles, thus indicating a leveling off in the capacity loss and an overall good longevity of MOF-LA2-1 (Supporting Information, Section S9).

**Figure 3:**
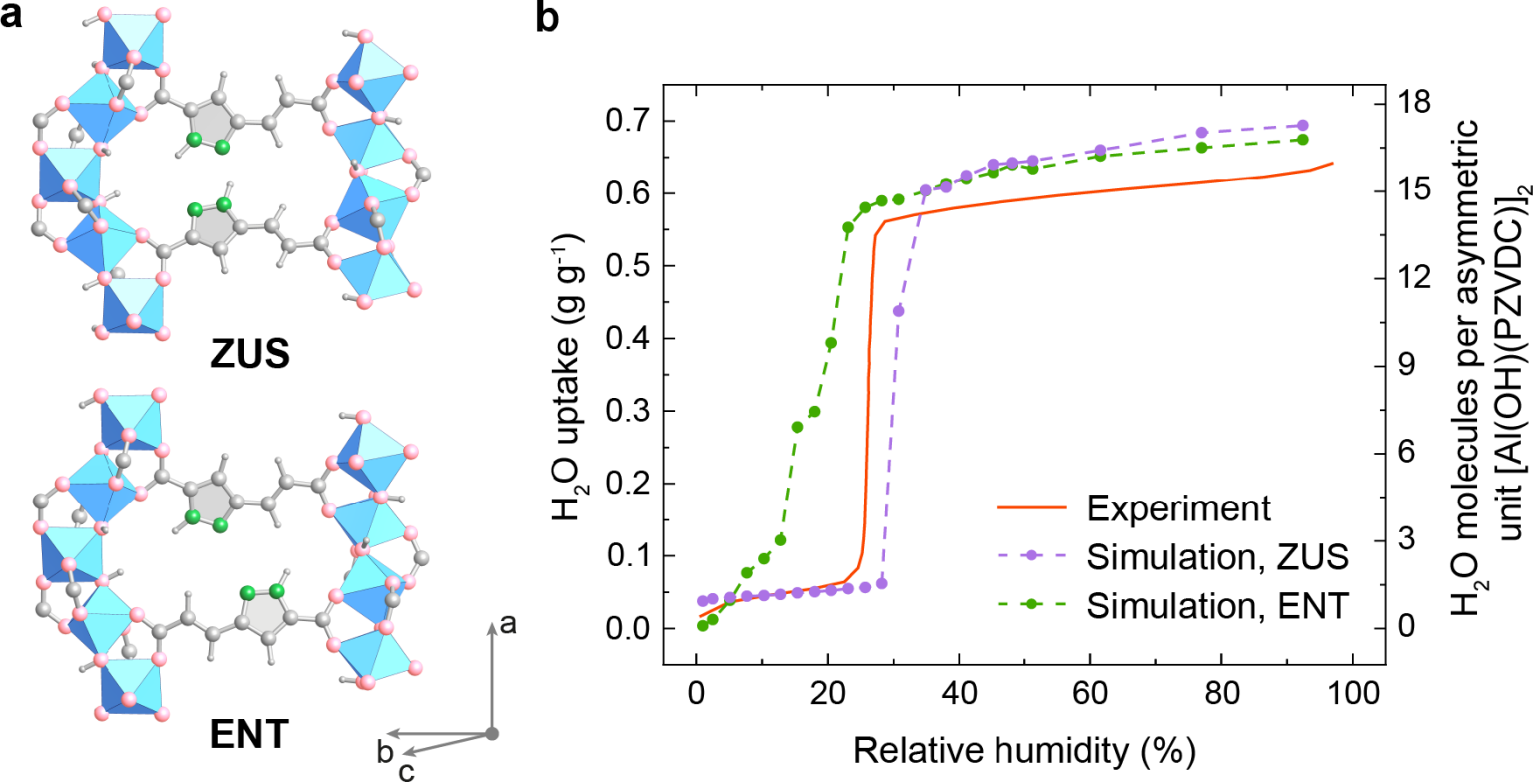
Water adsorption isotherms of MOF-LA2-1 exhibiting different linker configurations. (a) Most stable ZUS and ENT linker configurations utilized for the simulation. Coordinate system is given for guidance. Al, blue octahedron; C and H, gray; N, green; O, pink. (b) Adsorption isotherms were computed using GEMC simulations at 298 K. The simulated and experimental data are shown as dashed and solid lines, respectively.

Next, we studied the dependence of the water sorption behavior on the different linker configurations of MOF-LA2-1. This was accomplished by using force-field-based GEMC simulations to compute the water adsorption isotherms at 298 K (Supporting Information, Section S3.4) and water desorption isobars at water vapor pressures of 1.27 and 1.70 kPa (Supporting Information, Section S3.5). We focused these efforts on the most stable ZUS and ENT configurations, which served as representative examples of the different structural ensembles (Supporting Information, Section S3.4). The simulated water sorption isotherms of the two structural types displayed significantly different profiles (Figure 3): In good agreement with the measured adsorption isotherm, the ZUS configuration showed an initial water uptake of approximately one water molecule per asymmetric unit at 5% RH and a sharp isotherm step at 30% RH. In contrast, the computed isotherm of MOF-LA2-1 in the ENT configuration exhibited a more gradual profile, which could be explained by a greater number of water adsorption sites of varying binding strengths with the pore walls. Additionally, the experimental and simulated isobars for the ZUS configuration exhibited similar profiles, and the computed data suggested a downward shift of the inflection point by 13 °C compared to MOF-303, thus closely agreeing with the experimental data. On the contrary, the ENT configuration displayed a larger slope in the desorption curve at temperatures above 40 °C and a downward desorption temperature shift of only 9 °C compared to MOF-303 (Figure S8). Based on the comparison with the measured water sorption isotherm and isobars, it can be concluded that the simulated water sorption properties of the ZUS configuration are more representative of the experimental data, thus further supporting our structural model (Figure 1e).

In conclusion, we have demonstrated a linker “arm” extension strategy and employed it to significantly enhance the water-harvesting properties of the state-of-the-art water-harvesting material MOF-303. Importantly, MOF-LA2-1 with the extended linker features an approximately 50% increase of the water uptake capacity as well as reduced operational energetic requirements, while retaining the ability for moisture capture in arid regions and the hydrothermal stability suitable for long-term uptake and release cycling.

## Supporting Information Available

Additional descriptions of general methods and materials, synthetic procedures, computational studies, scanning electron microscopy, crystallography, powder X-ray diffraction analysis, thermogravimetric analysis, as well as nitrogen and water sorption analysis (PDF)
Machine-readable files for the DFT-optimized structures with different linker configurations of MOF-LA2-1, the DFT-optimized structures for the initial water adsorption sites in different configurations of MOF-LA2-1, the coordinates of the framework structures used in the GEMC simulations, and adsorption isotherm data in the adsorption isotherm file format (ZIP)
