# Supplementary material for: MOF Linker Extension Strategy for Enhanced Atmospheric Water Harvesting
Source: ACS Cent Sci. 2023 Mar 6;9(3):551–7. doi: 10.1021/acscentsci.3c00018 (PMC10037441; doi:10.1021/acscentsci.3c00018)
Supplement: Supplementary file 1 — oc3c00018_si_001.pdf [file oc3c00018_si_001.pdf]

Supporting Information

MOF Linker Extension Strategy for Enhanced Atmospheric Water Harvesting

Nikita Hanikel,[†,‡,#] Daria Kurandina,[†,‡,#] Saumil Chheda,[¤,#] Zhiling Zheng,[†,‡,§] Zichao Rong,[†,‡,§] S. Ephraim Neumann,[†,‡] Joachim Sauer,[&] J. Ilja Siepmann,[¤,*] Laura Gagliardi,[¥,*] Omar M. Yaghi[†,‡,§,ǁ,*]

[†]Department of Chemistry, University of California, Berkeley, CA 94720, United States

[‡]Kavli Energy Nanoscience Institute, University of California, Berkeley, CA 94720, United States

[¤]Department of Chemical Engineering and Materials Science, Department of Chemistry, and Chemical Theory Center, University of Minnesota-Twin Cities, Minneapolis, MN 55455, United States

[§]Bakar Institute of Digital Materials for the Planet, Division of Computing, Data Science, and Society, University of California, Berkeley, CA 94720, United States

[&]Institut für Chemie, Humboldt-Universität zu Berlin, 10099 Berlin, Germany

[¥]Department of Chemistry, Pritzker School of Molecular Engineering, and Chicago Center for Theoretical Chemistry, University of Chicago, Chicago, IL 60637, United States

[ǁ]KACST–UC Berkeley Center of Excellence for Nanomaterials for Clean Energy Applications, King Abdulaziz City for Science and Technology, Riyadh 11442, Saudi Arabia

[#]N.H., D.K., and S.C. contributed equally.

*Corresponding authors. Email: siepmann@umn.edu; lgagliardi@uchicago.edu; yaghi@berkeley.edu

Table of Contents

Section S1. General Methods and Materials 3

Section S2. Synthetic Procedures 8

Section S3. Computational Study of MOF-LA2-1 11

Section S3.1. Initial Prediction of the Pore Volume and Water Adsorption Properties 11

Section S3.2. Stability of Different Linker Configurations in MOF-LA2-1 12

Section S3.3. Determination of the Primary Water Adsorption Sites 17

Section S3.4. Simulation of Water Adsorption Isotherms 20

Section S3.5. Simulation of Water Desorption Isobars 23

Section S4. Scanning Electron Microscopy 26

Section S5. Single-Crystal X-Ray Diffraction Analysis 27

Section S6. Powder X-Ray Diffraction Analysis 28

Section S7. Thermogravimetric Analysis 31

Section S8. Nitrogen Sorption Analysis 32

Section S9. Water Sorption Analysis 35

Section S10. References 38

Section S1. General Methods and Materials

Chemicals:

All chemicals were purchased from commercial sources and used without additional purification. Ethyl 5-formyl-1*H*-pyrazole-3-carboxylate (**1**) was purchased from Enamine. *N*,*N*-dimethylformamide (DMF), methanol, acetone, hexane, tetrahydrofuran (THF) and methyl (triphenylphosphoranylidene)acetate (**2**) were purchased from Sigma-Aldrich. Ultrahigh-purity-grade (UHP-grade) N_2, He, and Ar (purity 99.999%), as well as ultra-zero-grade air were purchased from Praxair.

Analytical Techniques:

The powder X-ray diffraction (PXRD) and water sorption data of MOF-303 presented in this manuscript were extracted from previous publications.[1,2]

Liquid-state ^{1}H and ^{13}C NMR spectra were acquired on a Bruker NEO-500 (500 MHz). ^{1}H signals are referenced to residual $CHCl_3$ at 7.26 ppm or DMSO at 2.50 ppm. ^{13}C signals are referenced to $CDCl_3$ at 77.16 ppm or DMSO-d_6 at 39.52 ppm.

Elemental analysis measurements were performed using a Perkin Elmer 2400 Series II CHNS elemental analyzer.

PXRD analysis of MOF-LA2-1 obtained through solvothermal synthesis was conducted on a Bruker D8 Advance X-ray diffractometer equipped with a Cu anode and a Ni filter (CuKα radiation) in Bragg-Brentano geometry. The sample was mounted on a zero-background holder and leveled with a spatula. The PXRD patterns were recorded between 3 and 50° with 2303 steps (~0.02° per step) with an acquisition time of 10 seconds per step, thus resulting in ~6.5 hours analysis time.

The PXRD pattern of MOF-LA2-1 obtained through the green synthesis method was measured by using a Rigaku MiniFlex 6G equipped with a HyPix-400MF Hybrid Pixel Array detector and a normal-focus X-ray tube with a CuKα source. The zero-background holder is made of single crystal Si cut on a 310 axis. The PXRD patterns were recorded between 2 and 50° with 4801 steps (~0.01° per step) with scan speed of 0.5° per minute, thus resulting in ~1.5 hours analysis time per measurement.

Scanning electron microscopy images were obtained on an FEI Quanta 3D FEG scanning electron microscope with an accelerating voltage of 15 kV and a working distance of 10 mm. The samples were dispersed on carbon tape on a stainless-steel holder. Energy-dispersive X-ray spectroscopy (EDS) data were collected using an Oxford X-Max EDS system working at an acceleration voltage of 15 kV.

Optical microscope images were taken by using an HRX-01 digital microscope operated in the transmission mode. The sample was dispersed on a glass slide and then placed on a motorized stage for imaging.

Single-crystal X-ray diffraction (SCXRD) measurements were conducted at the beamline 12.2.1 at the Advanced Light Source (Lawrence Berkeley National Laboratory, USA) using a radiation wavelength of $\lambda = 0.7288$ Å. The beamline was equipped with a PHOTON-II CMOS detector operating in shutterless mode and a Si(111) monochromator. For the measurement, the crystal was mounted on a Kapton® MiTeGen MicroMount™ in a minimal amount of Paratone® N oil and submerged in a cold gas stream generated by an Oxford Cryosystems 800 Series Cryostream. Raw data processing was carried out with the APEX3 software package.[3] The data were integrated by using SAINT[4] and corrected for absorption with SADABS.[5] The structural solutions were determined by using intrinsic phasing (SHELXT)[6] and refined by the principle of least squares (SHELXL).[7] Both solution and refinement, as well as visualization of the electron density and the associated structural model were conducted by using the Olex2 software package.[8]

The TGA curves were recorded on a Netzsch Jupiter, STA 449 F5 apparatus. Prior to the measurement, the samples were dried by heating to 150 °C at a rate of 1 °C min^{-1}. The measurement was then initiated after the temperature in the TGA oven decreased to 40 °C. For the TGA measurement, the temperature was ramped from 40 to 800 °C at a heating rate of 1 °C min^{-1}. During the experiment, UHP-grade Ar at a flow rate of 60 mL min^{-1} was used for the balance purge flow; and UHP-grade Ar (inert conditions) or ultra-zero-grade air (oxidative conditions) at a flow rate of 60 mL min^{-1} was used for the sample purge flow.

Low-pressure nitrogen sorption measurements were carried out on a Micromeritics ASAP 2420 surface area analyzer. The N_2 isotherms were measured using a liquid nitrogen bath (77 K). UHP-grade He was used for free space corrections.

Water vapor sorption experiments were carried out on a BEL Japan BELSORP-aqua[3] or Micromeritics 3Flex Surface Characterization Analyzer. Prior to the measurements, the vapor source was degassed through five cycles freeze-pump-thaw. The measurement temperature was maintained in a water bath equipped with a thermostatic circulator. UHP-grade He was used for free space corrections.

The isobar measurements as well as the uptake and release cycling experiments were conducted with a TA Instruments DSC SDT Q600 Thermogravimetric Analyzer & Differential Scanning Calorimeter. The temperature and relative humidity (RH) were monitored using high-accuracy thermocouples and humidity sensors downstream of the TGA chamber. To adjust the RH in the TGA, a dry and a humidified N_2 feed were connected to the primary and secondary TGA inlet, respectively, and their flow rate ratio was varied according to a pre-recorded calibration curve, while maintaining the overall sum of both flow rates constant at 200 mL min^{-1}. UHP-grade N_2 was utilized as the dry N_2 feed. To generate the humidified N_2 feed, UHP-grade N_2, regulated by the Sierra SmartTrak® 100 mass flow controller, was passed through a H_2O-filled 2-L gas washing bottle, which was refilled frequently to ensure steady humidification.

Computational Methods:

Periodic density functional theory (DFT) calculations were performed on a single unit cell of MOF-LA2-1, containing eight formula units {[Al(OH)(PZVDC)], where PZVDC^{2-} = (*E*)-5-(2-carboxylatovinyl)-1*H*-pyrazole-3-carboxylate}, using the *Vienna Ab Initio Simulation Package* (VASP 6.2.1)[9] to compare the relative stability of MOF-LA2-1 structures exhibiting different linker configurations and to compute the binding energies of water molecules adsorbed at the primary sites of the framework. The Perdew-Burke-Ernzerhof (PBE)[10] exchange-correlation density functional was used along with Grimme's D3 dispersion correction[11] with Becke-Johnson damping. A plane-wave basis set with a kinetic energy cut-off of 850 eV was used for the geometry optimizations. A hard pseudopotential (ENMAX = 700 eV) was used for O, N, C, and H atoms, while a normal pseudopotential (ENMAX = 240.3 eV) was used for Al. Energy and force convergence criteria of 10^{-8} eV and 0.01 eV Å^{-1}, respectively, were employed for all optimizations. Due to the sufficiently large size of the unit cell of MOF-LA2-1, only a 1 × 1 × 1 Γ-centered k-point grid was used for the Brillouin zone sampling.

The initial-guess structures of MOF-LA2-1, representing different linker configurations, were constrained to the experimental cell parameters and constructed from crystallographic data obtained for the water-loaded MOF-LA2-1 (see Section S5 for more details). Only the atomic coordinates of the framework were relaxed during the geometry optimization without relaxing the shape or volume of the unit cell.

Low-energy MOF-LA2-1 structures with different linker configurations were then selected to investigate the most favorable water adsorption sites and compare these adsorption environments to that in MOF-303. The initial guess structures for water molecules adsorbed on different framework sites were generated based on chemical intuition and paralleling the known adsorption sites in analogous MOF-303. Both the atomic coordinates and the cell parameters were relaxed during the geometry optimization of the structure similar to our previous approach to determine the water adsorption sites in MOF-303.[1] The adsorption strength of the different sites was determined based on the average binding energy of the water molecules calculated as

$$\Delta E_{\mathrm{n}} = \frac{E_{\mathrm{MOF+water}} - E_{\mathrm{MOF}} - n \cdot E_{\mathrm{water,gas}}}{n}$$

where ΔE_{n} is the average binding energy per water molecule at a loading of n water molecules per unit cell, $E_{\mathrm{MOF+water}}$ is the combined electronic energy of the MOF and the adsorbed water molecules, and E_{MOF} and $E_{\mathrm{water,gas}}$ are the electronic energies of the reference pristine MOF and an isolated water molecule, respectively.

Force-field-based Monte Carlo (MC) simulations in the isobaric–isothermal Gibbs ensemble[12,13] were performed using the *Monte Carlo for Complex Chemical Systems - Minnesota* (MCCCS-MN) simulation software[14] to investigate water adsorption behavior in selected low-energy MOF-LA2-1 structures at $T = 298$ K. A simulation setup similar to that used previously for investigating the unary-vapor phase water adsorption in analogous MOFs, MOF-303 and MOF-333 was used.[15] The simulation setup consists of two simulation boxes, one for the MOF phase and one for the water gas-phase reservoir pre-equilibrated to $T = 298$ K and P_{target}, held in thermodynamic contact. A $3 \times 2 \times 2$ supercell of the MOF structure was used and kept rigid throughout the simulation. Rigid-body translation and rotation moves were performed on randomly selected water molecules to maintain thermal equilibrium. Configurational-bias swap moves allowed for transfer of the water molecules between the two simulation boxes to maintain chemical equilibrium. Volume moves

performed on the cubic reservoir box were used to maintain the target pressure. In a typical simulation, 1%, 39%, 30%, and 30% of the total moves were distributed into volume moves, swap moves, translation moves, and rotation moves, respectively.

The MOF–water and water–water interactions were described using non-polarizable force fields. For the water molecules, the rigid 4-site TIP4P model (saturated vapor pressure, P_{sat} = 4.54 ± 0.12 kPa at 298 K) was used.[16] The non-bonded Lennard-Jones (LJ) interaction parameters and partial charges for MOF-LA2-1 were derived from the force field used for MOF-303.[15] Particularly, the non-bonded parameters for the aluminum oxide rod, and the pyrazole and carboxylate groups of the linker were kept the same as that for MOF-303. The parameters for the -CH=CH- group were taken from the TraPPE-UA force field for butadienes.[17] The Lorentz-Berthelot mixing rules were used to determine the unlike LJ parameters for the MOF–water LJ interactions. A spherical cutoff at 14 Å was used for truncating the pairwise LJ and real-space Coulomb interactions. Analytical tail corrections and the Ewald summation method were employed for the long-range LJ and Coulomb interactions.

A total of N = 2000 water molecules were used for the adsorption simulations conducted at T = 298 K and P/P_{sat} = 0.01–1.0. Each adsorption simulation was started from the empty MOF structure—the MOF structure was optimized via DFT calculations in the presence of water molecules corresponding to a given loading, and such water molecules were deleted before the adsorption simulation—similar to the previous approach to simulate accurate water adsorption isotherms in MOF-303.[15] Most simulations were equilibrated for 50,000 MC cycles (1 MC cycle consisted of N = 2000 Monte Carlo moves), while much longer equilibration periods were used in the vicinity of the sharp step in the isotherm. At least another 50,000 MC cycles were used for the production period. To determine the statistical uncertainties, the production period was divided into 4 equal blocks.

Section S2. Synthetic Procedures

No unexpected or unusually high safety hazards were encountered while performing the synthetic procedures described in the following.

Synthesis of (*E*)-5-(2-carboxyvinyl)-1*H*-pyrazole-3-carboxylic acid (H_2PZVDC):

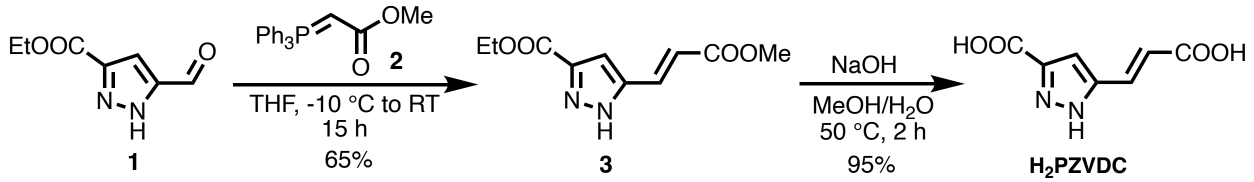

<u>*Step 1:*</u> A 100-mL round-bottom flask equipped with a stirring bar was charged with **1** (1.5 g, 8.9 mmol, 1 equiv.) and dry THF (50 mL) under argon atmosphere. The mixture was cooled down to –10 °C using an acetone/ice bath, and **2** (3.5 g, 10.5 mmol, 1.2 equiv.) was added portion-wise. The reaction was allowed to warm up to room temperature over a period of 15 hours. After concentrating the resulting solution under reduced pressure, a mixture containing *E*- and *Z*-isomers was identified via ^{1}H NMR analysis. The desired *E*-isomer **3** was isolated via column chromatography using acetone/hexane (1:5) as eluent (R_f = 0.1). Yield: 1.3 g, 65%.

^{1}H NMR (400 MHz, $CDCl_3$) δ 10.93 (s, 1H), 7.67 (d, J = 16.0 Hz, 1H), 7.04 (s, 1H), 6.48 (d, J = 16.0 Hz, 1H), 4.41 (q, J = 7.1 Hz, 2H), 3.81 (s, 3H), 1.41 (t, J = 7.1 Hz, 3H).

<u>*Step 2:*</u> A 100-mL round-bottom flask equipped with a stirring bar was charged with **3** (1.3 g, 5.8 mmol, 1 equiv.), MeOH (50 mL) and aqueous NaOH solution (20 mL, 1.5 M, 5 equiv.). The reaction was heated at 50 °C (oil bath temperature) until the starting material was consumed, as monitored by TLC (2 hours). The solution was concentrated under reduced pressure and 5 M HCl was added dropwise until pH 2–3. The resulting precipitate was filtered off and thoroughly washed with H_2O (4 × 10 mL) and MeOH (1 × 5 mL). After drying at 50 °C in vacuo, the linker H_2PZVDC was obtained as white powder. Yield: 1.0 g, 95%.

^{1}H NMR (500 MHz, DMSO-d_6) δ 13.80–13.10 (br. s, 3H), 7.46 (d, J = 16.2 Hz, 1H), 7.17 (s, 1H), 6.53 (d, J = 16.1 Hz, 1H). ^{13}C NMR (126 MHz, DMSO-d_6) δ 167.4, 161.4, 120.6, 108.4. HRMS (m/z): $[M-H]^-$ calcd. for $C_7H_5N_2O_4$, 181.0255; found, 181.0255.

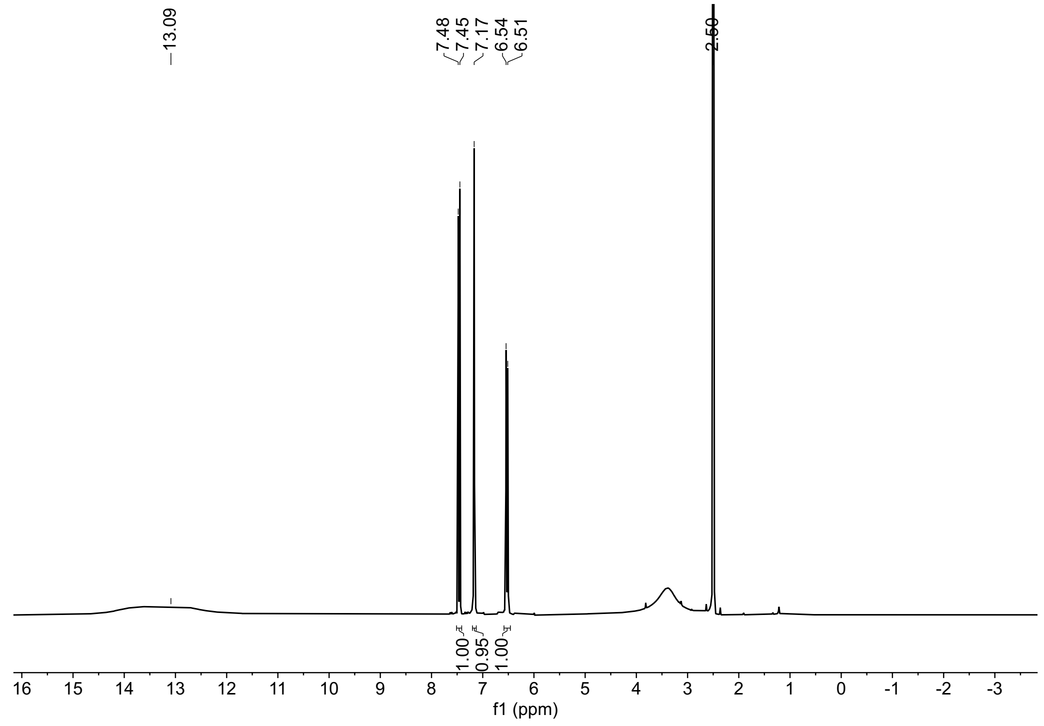

Figure S1. ^{1}H NMR spectrum of H_2PZVDC (500 MHz, DMSO-d_6).

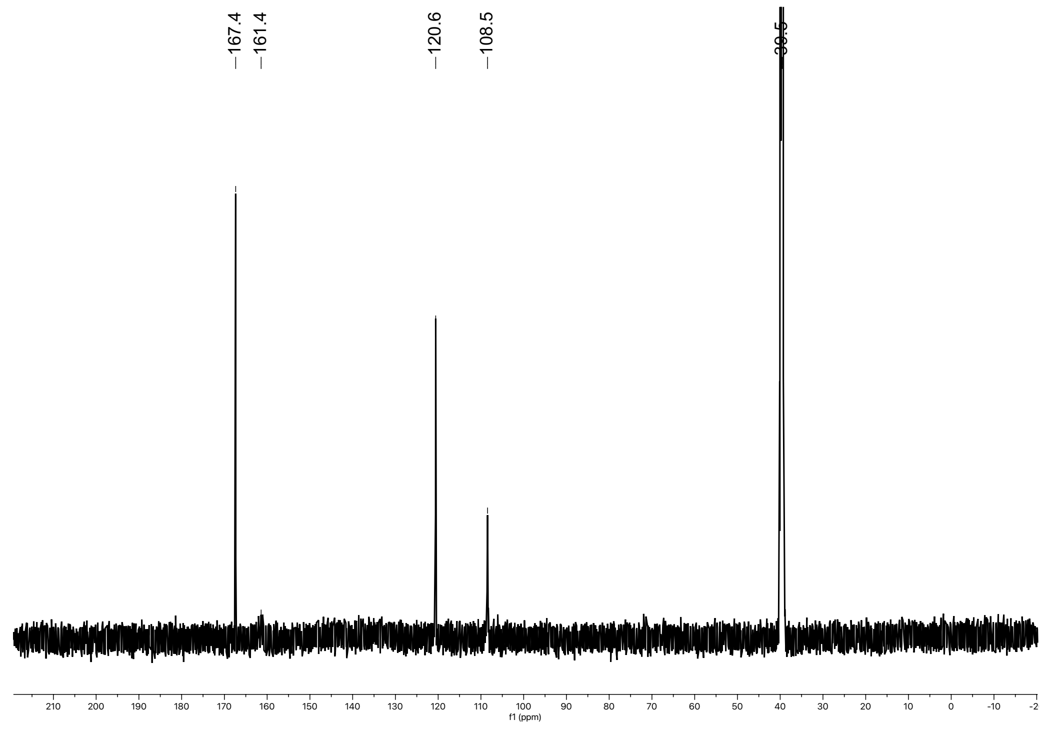

Figure S2. ^{13}C NMR spectrum of H_2PZVDC (126 MHz, DMSO-d_6).

Solvothermal synthesis of MOF-LA2-1:

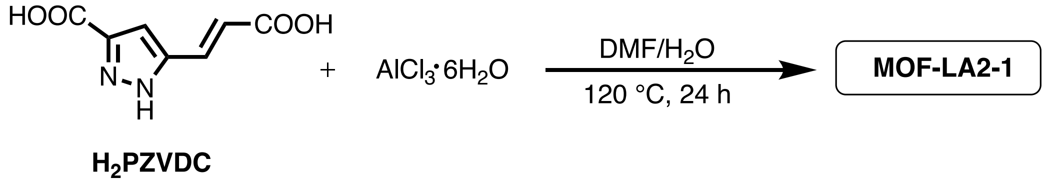

In a 4-mL scintillation vial, the linker H_2PZVDC (91.0 mg, 0.5 mmol, 1 equiv.) was dissolved in *N*,*N*-dimethylformamide (DMF) (0.6 mL) upon sonication. An aqueous solution of $AlCl_3 \cdot 6H_2O$ (2.4 mL, 0.2 M, 1 equiv.) was added dropwise, and the resulting mixture was heated in a 120 °C oven for 24 hours. After cooling down to room temperature, the white precipitate was collected by centrifugation and washed with H_2O (3 × 30 mL) and MeOH (3 × 30 mL). MOF-LA2-1 was activated under dynamic vacuum (~10^{-3} mbar) for 12 hours at room temperature, followed by gradual heating to 120 °C for 6.5 hours. Yield: 65.0 mg, 58%.

Elem. Anal. of MOF-LA2-1: Calcd. for $C_{56}H_{40}N_{16}O_{40}Al_8$: C, 37.52; H, 2.25; N, 12.50%. Found: C, 36.78; H, 2.38; N, 11.95%.

Green synthesis of MOF-LA2-1:

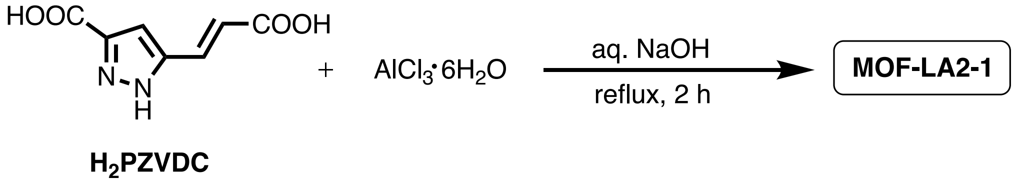

In a 50-mL round-bottom flask, the linker H_2PZVDC (364 mg, 2 mmol, 1 equiv.) and NaOH (160 mg, 4 mmol, 2 equiv.) were dissolved in deionized water (10 mL) upon sonication. An aqueous solution of $AlCl_3 \cdot 6H_2O$ (6 mL, 0.33 M, 1 equiv.) was added dropwise for 10 minutes, and the reaction mixture was heated to 120 °C and refluxed for 2 hours. After cooling down to room temperature, the white powder was collected by centrifuging and washed with deionized water (2 × 10 mL) and EtOH (3 × 10 mL). The white powder was dried under air for 12 hours, followed by activation under dynamic vacuum (~10^{-3} mbar) for 12 hours at 120 °C. Yield: 301 mg, 66%.

Elem. Anal. of MOF-LA2-1: Calcd. for $C_{56}H_{40}N_{16}O_{40}Al_8$: C, 37.52; H, 2.25; N, 12.50%. Found: C, 37.29; H, 2.43; N, 12.10%.

Section S3. Computational Study of MOF-LA2-1

Section S3.1. Initial Prediction of the Pore Volume and Water Adsorption Properties

We first constructed a hypothetical MOF, MOF-LA2-1-FO (FO, full optimization), from the parent MOF-303 wherein the $PZDC^{2-}$ (1*H*-pyrazole-3,5-dicarboxylate) linkers of MOF-303 were replaced with $PZVDC^{2-}$ linkers containing an extension by a vinyl group. Without any *a priori* knowledge of the crystal structure of this MOF, we constructed a DFT-optimized structure of this MOF, where the contact angle between the aluminum oxide rods and linkers was similar to that in MOF-303 with the pyrazole moieties forming an alternating pattern of hydrophilic–hydrophobic pockets. In this arrangement, the vinyl group extension allowed for a more than 30% increase in pore volume compared to the parent MOF (0.598 cm^3 g^{-1} versus 0.452 cm^3 g^{-1}). Force-field-based Monte Carlo simulations in the isobaric-isothermal Gibbs ensemble (see Section S1, *Computational Methods* for more details) were used to predict the water adsorption isotherm of MOF-LA2-1-FO at 298 K (Figure S3). The simulated adsorption isotherm for MOF-LA2-1-FO showed a steep step at a relative humidity of ~18% and an overall water uptake of 0.6 g g^{-1}—a 1.5-fold increase compared to the uptake of MOF-303-FO that was generated by using the same procedure. It should be noted that the position of the step and the saturation loading for MOF-LA2-1-FO are shifted to lower values compared to the simulations for MOF-LA2-1 obtained from partial optimization of atomic coordinates while retaining the experimentally determined lattice parameters (Section S3.4).

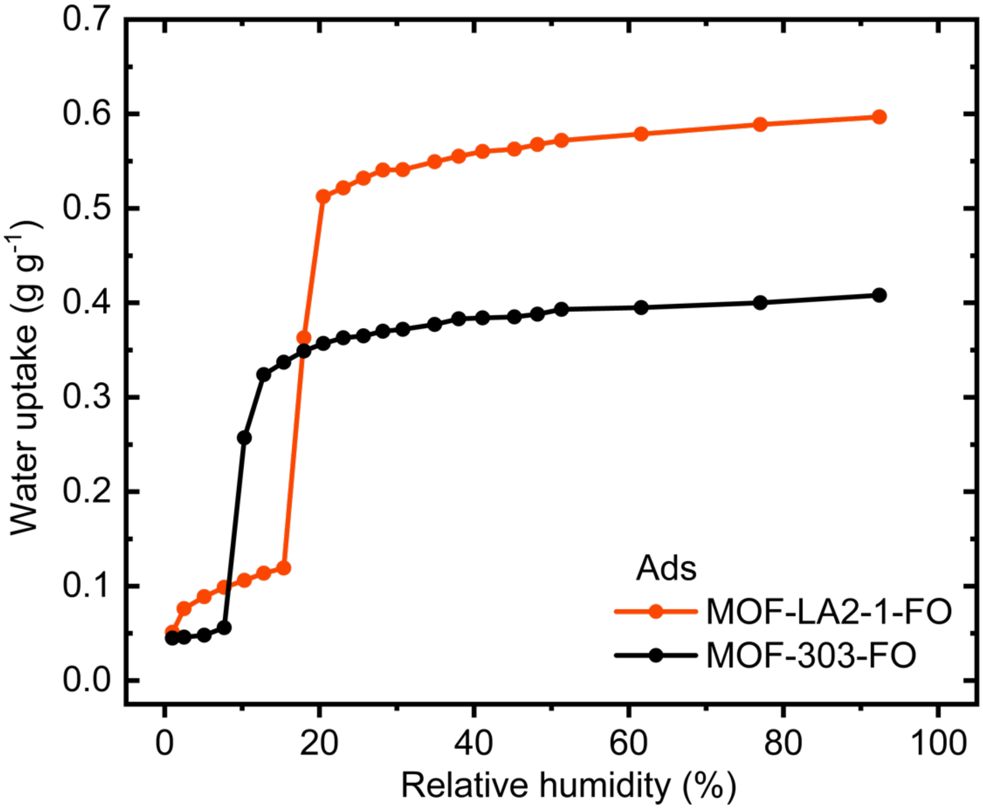

Figure S3. Water adsorption isotherms of MOF-LA2-1-FO and MOF-303-FO simulated at 298 K using force-field-based Gibbs ensemble Monte Carlo (GEMC) simulations. Rigid framework structures were used for both MOFs, which were DFT optimized (full optimization including both atomic coordinates and cell parameters) in the presence of four water molecules per unit cell. The respective water molecules were deleted prior to the GEMC simulations.

Section S3.2. Stability of Different Linker Configurations in MOF-LA2-1

The exact linker configuration(s) of MOF-LA2-1 could not be determined experimentally, which could be associated with positional disorder of the asymmetric linker in the crystal structure. Accordingly, we utilized DFT calculations to probe the relative stability of the different possible linker configurations in the MOF-LA2-1 structure. A total of 16 different linker configurations varying in the position and orientation of the pyrazole and vinyl groups in the hydrophilic cavity of the MOF were considered. We use a four-part labeling convention for these different linker configurations, which are depicted for classification purposes such that the wide side of the pocket is on the left and the symmetry equivalent μ_2-OH groups (labeled as 2; Figure S4) are on the bottom left and right side of the pocket. The first part of the naming convention indicates whether the

pyrazole rings from the opposite linkers in the hydrophilic pocket of the MOF are on the same side {denoted as ZUS (from German '*zusammen',* together)} or on alternate sides {denoted as ENT (from German '*entgegen',* opposite)} of the cavity. The second part of the naming convention indicates if the pyrazole ring at the top of the cut-away view is located on the wide (denoted as w) or narrow (denoted as n) side of the pocket. Lastly, the geometries of the vinyl groups with respect to the corresponding pyrazole rings are reflected by the *cis/trans* notation starting with the linker on the top.

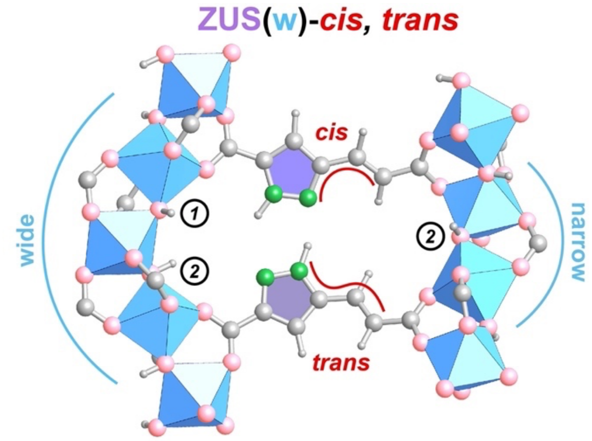

Figure S4. Illustration of the four-part labeling convention serving to describe possible linker configurations in MOF-LA2-1. The μ_2-OH functionalities are labeled with Arabic numbers, indicating symmetry equivalent groups.

The 16 different linker configurations are illustrated in Figure S5. Their relative stabilities were evaluated using periodic DFT optimizations (see Section S1, *Computational Methods* for more details) of the framework atoms of the empty MOF constrained to the lattice parameters determined experimentally (see Section S5 for more details). In general, the ZUS linker configurations, in which the pyrazole rings are opposite to each other in the hydrophilic cavity of the MOF, were found to be more stable compared to the ENT linker configurations, which could be attributed to hydrogen-bond stabilization between the opposing pyrazole rings in the ZUS linker configurations ($H_{donor}\cdots N_{acceptor}$ distance of 1.85 Å and $N_{donor}–H_{donor}\cdots N_{acceptor}$ angle of 152° between opposite pyrazole N in the most stable ZUS linker configuration). In contrast to the ZUS linker configurations, in which the pyrazole rings in the hydrophilic MOF cavity were aligned in the same plane, the pyrazole rings in the hydrophilic cavity of the MOF with ENT linker configurations were not aligned in a common plane. Moreover, the ZUS linker configurations in

which the pyrazole rings were present on the wider side of the hydrophilic cavity {ZUS(w)} were found to be more stable compared to the linker configurations in which the pyrazole rings were present on the narrower side of the hydrophilic pocket {ZUS(n)}. This could be explained by potential steric constraints associated with both relatively large pyrazole moieties being present on the narrow side of the pocket. The orientation of the vinyl group was also found to influence the relative stability of the MOF-LA2-1 structures. Generally, the presence of *cis*-oriented vinyl groups relative to the pyrazoles in the ZUS(w) configurations destabilized the MOF structures. In contrast, the ZUS(n) configurations were stabilized by presence of *cis*-oriented vinyl groups.

To summarize, the ZUS(w)-*trans,trans* linker arrangement was found to be the most stable configuration of MOF-LA2-1. Four other linker configurations (namely ZUS(n)-*cis,trans*; ENT(w)-*trans,cis*; ZUS(w)-*trans,cis*; and ZUS(w)-*cis,trans*) were identified as energetically accessible linker configurations {with ΔE values of 27–29 $kJ \cdot mol^{-1}$ per asymmetric unit $[Al(OH)(PZVDC)]_2$, that is ~8 $k_B T$ at MOF synthesis temperatures of 373–393 K}. The first four structures were used as representative structures to investigate the water adsorption behavior of MOF-LA2-1 computationally, while calculations were not carried out for ZUS(w)-*cis*,*trans* due to its similarity to ZUS(w)-*trans*,*cis.*

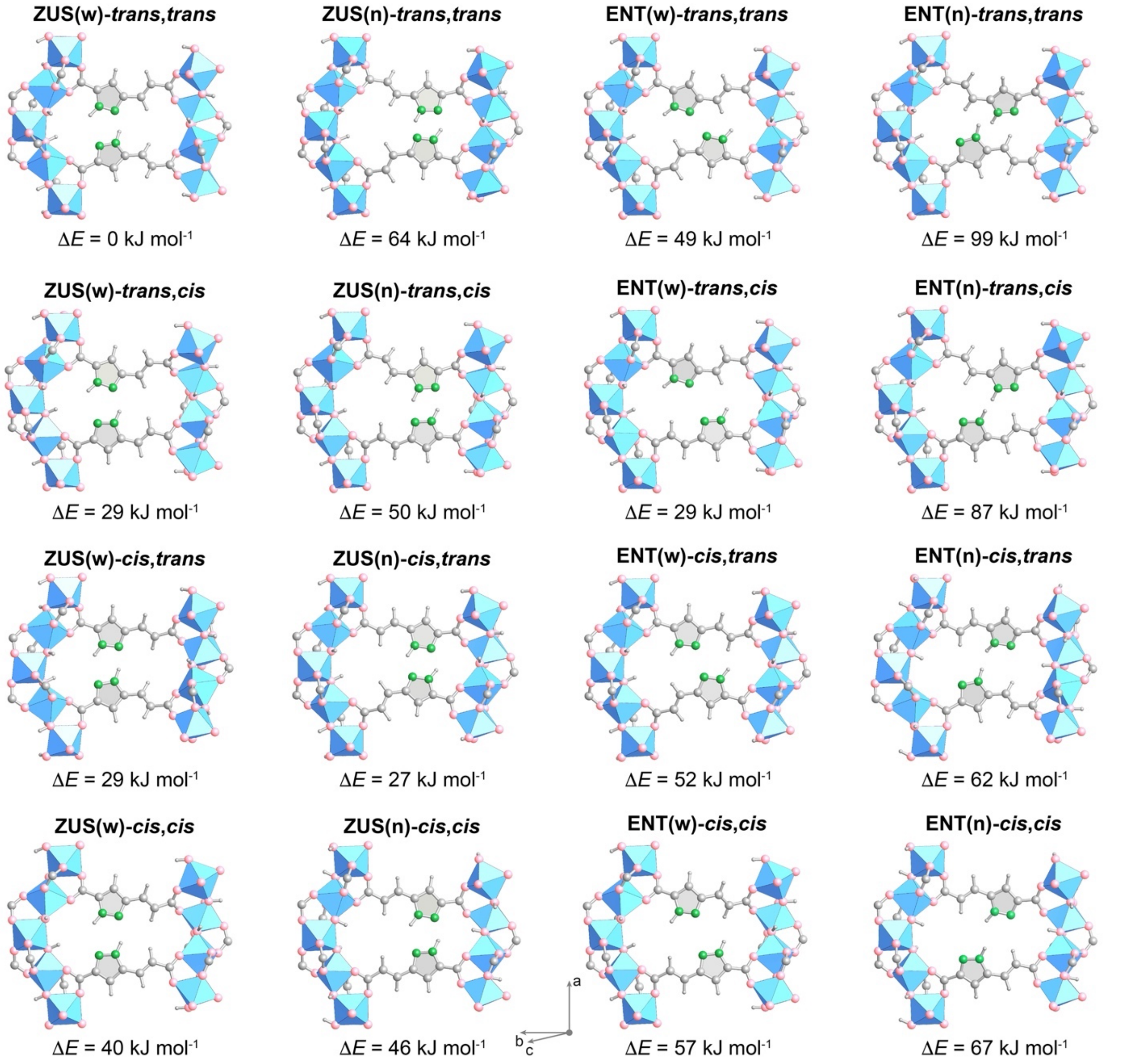

Figure S5. 16 linker configurations in MOF-LA2-1 optimized using DFT. The hydrophilic pocket of the MOF is displayed for each configuration, where the pyrazole rings are present on the same side of the hydrophilic cavity (ZUS; two columns on the left) or on alternate sides of the hydrophilic cavity (ENT; two columns on the right) with *trans*- or *cis*-orientations of the vinyl group with respect to the pyrazole rings. The electronic stabilities per asymmetric unit $[Al(OH)(PZVDC)]_2$ (ΔE) of the different MOF-LA2-1 structures relative to the ZUS(w)-*trans,trans* linker arrangement obtained from DFT calculations are provided underneath each structure. The coordinate system is given for guidance. Al, blue octahedron; O, pink; N, green; C and H, gray.

Table S1. Absolute electronic energies of a unit cell (*E* in Hartree) and relative electronic stabilities (ΔE in kJ mol^{-1}) per asymmetric unit $[Al(OH)(PZVDC)]_2$ relative to the ZUS(w)-*trans*,*trans* linker arrangement of the different MOF-LA2-1 structures obtained from DFT calculations. A unit cell of MOF-LA2-1 consists of 4 asymmetric units.

**Linker configuration**	***E* (Hartree)**	**ΔE (kJ mol^{-1})**
ZUS(w)-*trans*,*trans*	–1139.743262	0
ZUS(w)-*trans*,*cis*	–1138.537555	29
ZUS(w)-*cis*,*trans*	–1138.521767	29
ZUS(w)-*cis*,*cis*	–1138.080089	40
ZUS(n)-*trans*,*trans*	–1138.324908	64
ZUS(n)-*trans*,*cis*	–1137.660001	50
ZUS(n)-*cis*,*trans*	–1138.619317	27
ZUS(n)-*cis*,*cis*	–1137.821428	46
ENT(w)-*trans*,*trans*	–1137.697891	49
ENT(w)-*trans*,*cis*	–1138.559716	29
ENT(w)-*cis*,*trans*	–1137.604073	52
ENT(w)-*cis*,*cis*	–1137.383055	57
ENT(n)-*trans*,*trans*	–1135.656132	99
ENT(n)-*trans*,*cis*	–1136.116325	87
ENT(n)-*cis*,*trans*	–1137.191406	62
ENT(n)-*cis*,*cis*	–1136.947796	67

Section S3.3. Determination of the Primary Water Adsorption Sites

MOF-LA2-1 was derived from MOF-303 by adding a compact, yet long vinyl group to the $PZDC^{2-}$ linker of MOF-303 with the goal of enhancing the water uptake capacity of MOF-303 while retaining its arrangement of the pyrazole functionalities, which was determined to be key for the favorable water-harvesting properties of MOF-303.[1] To demonstrate this, we investigated the primary water adsorption sites in the ZUS(w)-*trans*,*trans* and ENT(w)-*trans*,*cis* linker configurations of MOF-LA2-1 (Figure S6), which served as representative structures for the ZUS and ENT configurations. Indeed, similar to the primary water adsorption sites determined previously in MOF-303 (Figure S6a,b), water molecules are adsorbed in sites constituted by pyrazole moieties as well as μ_2-OH groups of the aluminum oxide rods in both linker configurations.

In the ZUS(w)-*trans*,*trans* linker configuration, the first water molecule forms four H bonds (2.7–3.0 Å) with the framework—one each with the NH and N groups of the two neighboring linkers, and two with the μ_2-OH groups of the aluminum oxide rod ($\Delta E_{ads,avg}$ = –84.6 kJ mol^{-1}; Figure S6c). The second water molecule adsorbs through two H bonds (both 2.7 Å)—one each with the NH and N groups of the remaining, neighboring pyrazole moieties ($\Delta E_{ads,avg}$ = –57.7 kJ mol^{-1}; Figure S6d). These water adsorption sites are similar to those observed in MOF-303, where the first H_2O molecule adsorbs with a comparable strength and the second H_2O molecule adsorbs stronger than in MOF-LA2-1 {$\Delta E_{ads,avg}$ = –82.4 and –80.2 kJ mol^{-1} at 1 and 2 H_2O molecules per asymmetric unit $[Al(OH)(PZDC)]_2$, respectively; Figure S6a,b}. The subsequent H_2O molecule is anticipated to adsorb at the remaining μ_2-OH group of the Al oxide rod, as observed previously for MOF-303.[1]

In contrast, the H_2O adsorption sites differ in the ENT(w)-*trans*,*cis* linker configuration (Figure S6e,f), which could be explained by the spatial separation of the pyrazole rings. The first H_2O molecule adsorbs through four H bonds with the framework (2.7–2.9 Å)—one with the NH group, two with the μ_2-OH groups, and one with the carboxylate group of the linker ($\Delta E_{ads,avg}$ = –77.4 kJ mol^{-1}; Figure S6e). The second H_2O molecule adsorbs through two H bonds (2.7 and 3.0 Å)—one with the NH group and one with the μ_2-OH group of the Al oxide rod ($\Delta E_{ads,avg}$ = –63.9 kJ mol^{-1}; Figure S6f). We note that in this linker configuration, the N sites of

the linkers can adsorb subsequent water molecules, thereby leading to a higher number of favorable framework sites for H_2O adsorption compared to MOF-303.

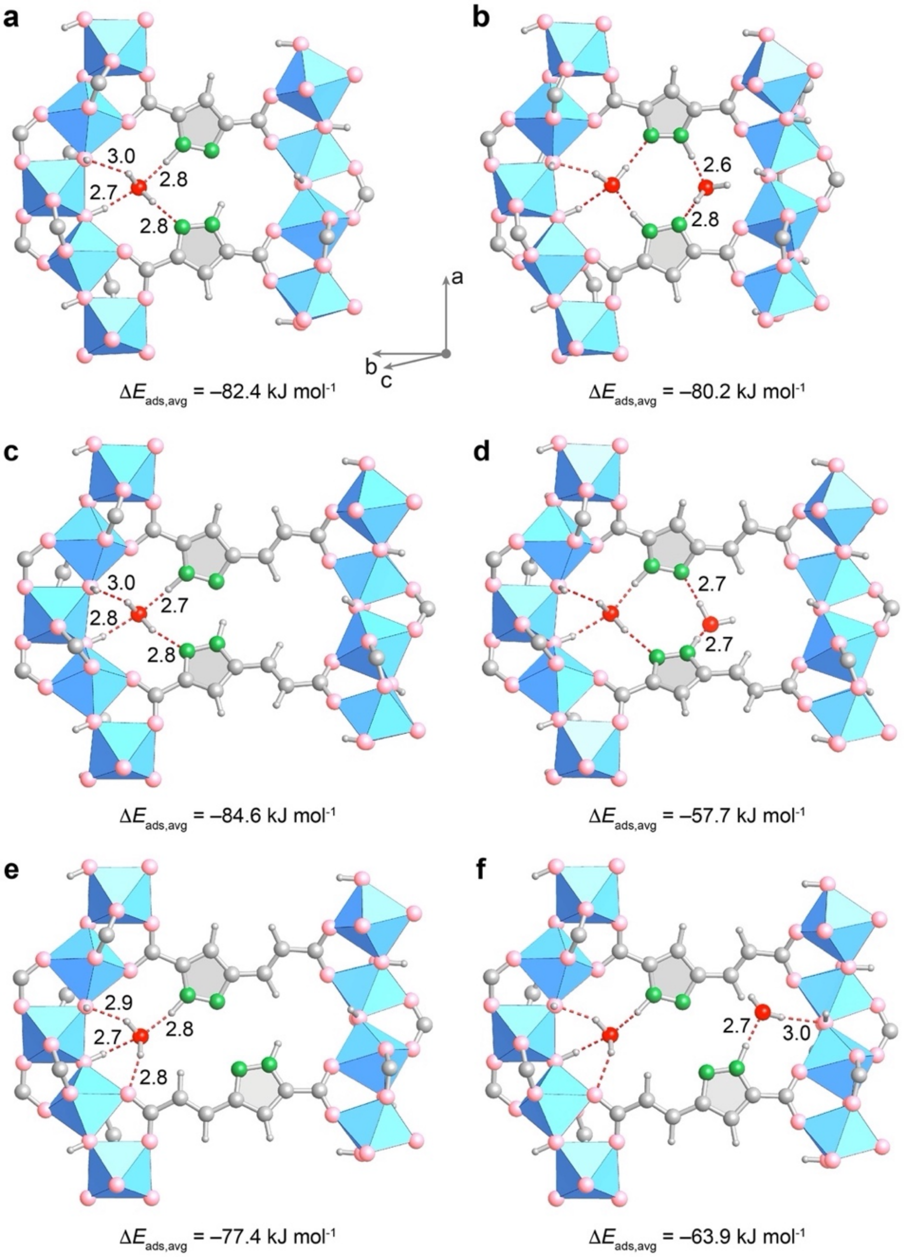

Figure S6. Primary water binding sites in MOF-303 (a,b), MOF-LA2-1 in the ZUS(w)-*trans*,*trans* configuration (c,d), and MOF-LA2-1 in the ENT(w)-*trans*,*cis* configuration (e,f) at water loadings of 1 (a,c,e) and 2 (b,d,f) H_2O molecules per asymmetric unit {$[Al(OH)(PZDC)]_2$ and $[Al(OH)(PZVDC)]_2$ in MOF-303 and MOF-LA2-1, respectively}. The average binding energy of water molecules ($\Delta E_{ads,avg}$) is reported in kJ mol^{-1}. The H-bond distances between the heteroatoms are given in Å. Al, blue octahedra; C and H, gray; N, green; O in framework, pink; O in H_2O, red.

Table S2. Absolute electronic energies of a unit cell (E in Hartree) and average binding energy ($\Delta E_{ads,avg}$ in kJ mol^{-1}) of a water molecule at water loadings of 1 and 2 H_2O molecules per asymmetric unit [Al(OH)(PZVDC)]$_2$ in the ZUS(w)-*trans*,*trans* and ENT(w)-*trans*,*cis* configurations obtained from full DFT optimization (atomic coordinates and cell parameters) of a unit cell of MOF-LA2-1. A unit cell of MOF-LA2-1 contains four asymmetric units and hence four equivalent water adsorption sites.

**Water loading per asymmetric unit**	***E* (Hartree)**	**$\Delta E_{ads,avg}$ (kJ mol^{-1})**
isolated water molecule (gas)	-14.291345	-
**ZUS(w)-*trans,trans***		
0	-1139.784378	0.0
1	-1200.458582	-84.6
2	-1258.902424	-57.7
**ENT(w)-*trans,cis***		
0	-1138.600536	0.0
1	-1198.973898	-77.4
2	-1258.227018	-63.9

Section S3.4. Simulation of Water Adsorption Isotherms

We next probed the dependence of the water adsorption behavior on the different linker configurations of MOF-LA2-1. Force-field-based Monte Carlo simulations in the isobaric–isothermal Gibbs ensemble (GEMC) were used to compute the water adsorption isotherms at 298 K. Considering the similarity of the primary adsorption sites in MOF-LA2-1 and MOF-303 (Section S3.3), the simulation setup was chosen to be similar to our previous study focusing on the prediction of water adsorption isotherms of MOF-303 (see Section S1, *Computational Methods* for more details).[15] Rigid framework structures of MOF-LA2-1, optimized in the presence of 1 H_2O molecule per asymmetric unit that were deleted prior to the GEMC simulations, were used for these calculations. This arrangement led to an expanded hydrophilic cavity, thus accounting for the structural flexibility of the MOF, which was previously shown to be important for obtaining an accurate initial water uptake in MOF-303.[15]

Using the above-described procedure, the water adsorption isotherms of MOF-LA2-1 in the ZUS(w)-*trans*,*trans*; ZUS(n)-*cis*,*trans*; ZUS(w)-*trans*,*cis*; and ENT(w)-*trans*,*cis* linker configurations were simulated (Figure S7). Noteworthy, the ZUS and ENT linker configurations exhibit significantly different water adsorption behavior. In agreement with the measured adsorption isotherm, both the ZUS(w)-*trans*,*trans* and ZUS(w)-*trans*,*cis* configurations, in which the pyrazole rings are present on the wider side of the hydrophilic cavity, show an initial water uptake of ~0.6–1 H_2O molecule per asymmetric unit at already 5% RH and a sharp step in the isotherm at 28–30% RH, slightly shifted compared to the experimental isotherm. We note that these two linker configurations differ only in the orientation of the vinyl groups, and the similar adsorption behavior of these two linker configurations suggests that the orientation of the vinyl groups (*cis* or *trans*) does not significantly influence the overall adsorption isotherm. On the other hand, the ZUS(n)-*cis*,*trans* linker configuration, in which the pyrazole rings are present on the narrowed side of the hydrophilic cavity, does not exhibit the initial water uptake at < 10% RH observed in the experimental isotherm, even though the framework structure used for this linker configuration was optimized in the presence of 1 H_2O molecule per asymmetric unit. This is consistent with the observation that the water molecules did not adsorb at the strong adsorption sites during the DFT optimization, as calculated for the other ZUS linker configurations. Instead, the adsorbed water molecules move out of the plane of the two pyrazole linkers into the MOF pore, thereby not expanding the cavity significantly upon water adsorption. This linker configuration

displayed a steep step in the isotherm at ~22% RH, thus exhibiting a larger deviation from the experimental isotherm than the ZUS(w) configurations.

In contrast to the steep step observed in the adsorption isotherms for the three investigated ZUS linker configurations, the ENT(w)-*trans*,*cis* linker configuration exhibited a more gradual increase in its water uptake. The pyrazole functionalities are more distributed across the hydrophilic cavity, leading to a greater number of energetically favorable adsorption sites in the framework compared to the ZUS linker configurations. Considering the steep profile of the experimental isotherm, we conclude that the ENT linker configuration is not a suitable structural model, while the ZUS(w)-*trans*,*trans* and ZUS(w)-*trans*,*cis* linker configurations appear to be good representatives of the synthesized MOF-LA2-1.

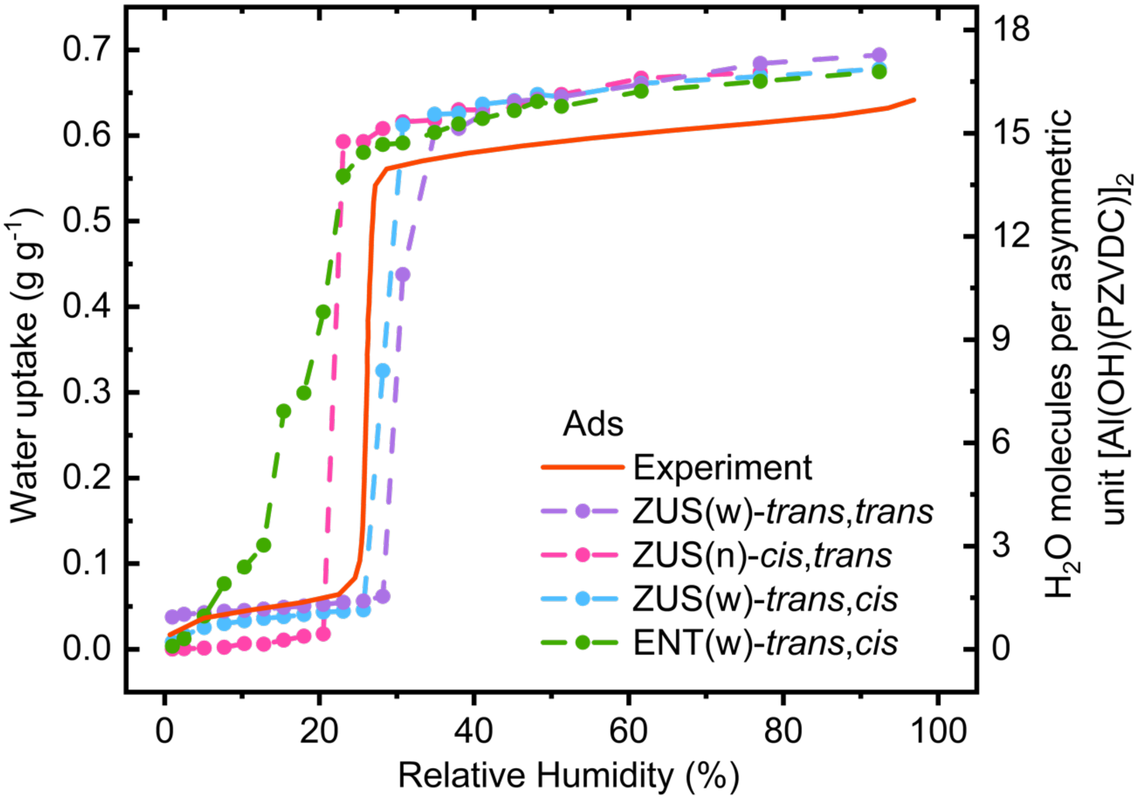

Figure S7. Simulated water adsorption isotherms (dashed) of the most stable linker configurations ZUS(w)-*trans*,*trans*; ZUS(n)-*cis*,*trans*; ZUS(w)-*trans*,*cis*; and ENT(w)-*trans*,*cis* along with the experimental isotherm (solid) of MOF-LA2-1. The saturated vapor pressure of water (TIP4P water model) at 298 K is $P_{sat} = 4.54 \pm 0.12$ kPa.

Table S3. Tabulated data for water adsorption isotherms calculated for MOF-LA2-1 in the ZUS(w)-*trans*,*trans*; ZUS(n)-*cis*,*tran*s; ZUS(w)-*trans*,*cis*; and ENT(w)-*trans*,*cis* linker configurations at 298 K using isobaric–isothermal GEMC simulations. The saturated vapor pressure of water (TIP4P water model) at 298 K is $P_{sat} = 4.54 \pm 0.12$ kPa. The water uptake is reported in g g^{-1}, where the subscripts denote the uncertainty in the last digit(s).

Relative Humidity (%)	Water uptake (g g^{-1})			
	**ZUS(w)-*trans,trans***	**ZUS(n)-*cis,trans***	**ZUS(w)-*trans,cis***	**ENT(w)-*trans,cis***
0.010	0.0377_{2}	0.0002_{1}	0.0084_{9}	0.0036_{2}
0.025	0.0407_{1}	0.0007_{2}	0.0166_{2}	0.012_{1}
0.051	0.0425_{1}	0.0014_{2}	0.0254_{16}	0.039_{1}
0.077	0.0443_{1}	0.0023_{3}	0.0300_{4}	0.077_{3}
0.103	0.0454_{1}	0.0067_{9}	0.0334_{7}	0.096_{1}
0.128	0.0468_{1}	0.006_{1}	0.0361_{6}	0.122_{3}
0.154	0.0489_{2}	0.0107_{6}	0.0380_{4}	0.278_{4}
0.180	0.0504_{1}	0.015_{1}	0.0406_{6}	0.299_{2}
0.205	0.0524_{1}	0.018_{1}	0.0434_{6}	0.394_{3}
0.231	0.0548_{2}	-	0.0444_{6}	0.553_{5}
0.257	0.0564_{5}	0.593_{3}	0.0459_{6}	0.580_{1}
0.282	0.062_{3}	0.593_{4}	0.325_{38}	0.589_{1}
0.308	0.438_{8}	0.608_{2}	0.612_{1}	0.591_{2}
0.349	0.604_{3}	0.616_{2}	0.625_{3}	0.604_{3}
0.380	0.608_{2}	0.618_{2}	0.6260_{6}	0.614_{3}
0.411	0.624_{2}	0.630_{3}	0.636_{1}	0.620_{1}
0.452	0.640_{1}	0.630_{1}	0.641_{2}	0.629_{2}
0.482	0.642_{4}	0.633_{1}	0.648_{1}	0.640_{2}
0.513	0.645_{2}	0.641_{1}	0.645_{2}	0.634_{3}
0.616	0.660_{3}	0.648_{2}	0.661_{2}	0.652_{2}
0.770	0.684_{3}	0.667_{1}	0.6690_{7}	0.663_{1}
0.924	0.694_{1}	0.674_{1}	0.6778_{5}	0.675_{2}

Section S3.5. Simulation of Water Desorption Isobars

We also probed the dependence of the water desorption behavior on the different linker configurations of MOF-LA2-1. Force-field-based GEMC simulations in the isobaric–isothermal ensemble (see Section S3.4) were used to compute the water loading at water vapor pressures of 1.27 and 1.70 kPa and temperatures ranging from 25 to 70 °C. The desorption simulations were carried out for MOF-303, as well as for MOF-LA2-1 in the ZUS(w)-*trans*,*trans* and ENT(w)-*trans*,*cis* linker configurations (Figure S8). Noteworthy, the ratio between the saturated vapor pressure of the TIP4P model and the experimental data is slightly temperature dependent. Nevertheless, the desorption simulations were carried out at the same two pressures as the experimental data, and no attempt was made to match the RH values at the different temperatures. Furthermore, we note that the TIP4P model overestimates the vapor pressure by a factor of 1.43 at 25 °C. Thus, the simulated desorption curves are shifted to lower temperatures (about 6 °C) compared to the experimental data (Figure 2c).

More importantly, the simulations yielded a downward shift of the inflection point for the desorption curve of MOF-LA2-1 in the ZUS(w)-*trans*,*trans* linker configuration compared to MOF-303 by 12 and 13 °C at 1.27 and 1.70 kPa, respectively. This shift agrees well with the 13 °C difference observed experimentally, and the profiles of the simulated desorption isobars of MOF-LA2-1 in the ZUS(w)-*trans*,*trans* linker configuration and MOF-303 match their experimental counterparts. In contrast, as expected from the more gradual increase in adsorption loading (see Figure S7), MOF-LA2-1 in the ENT(w)-*trans*,*cis* linker configuration yielded a larger slope of the desorption curve at temperatures above 40 °C compared to the simulation for the ZUS(w)-*trans*,*trans* linker configuration and the experiment. Furthermore, the downward temperature shift is only 9 °C for the ENT(w)-*trans*,*cis* linker configuration compared to MOF-303.

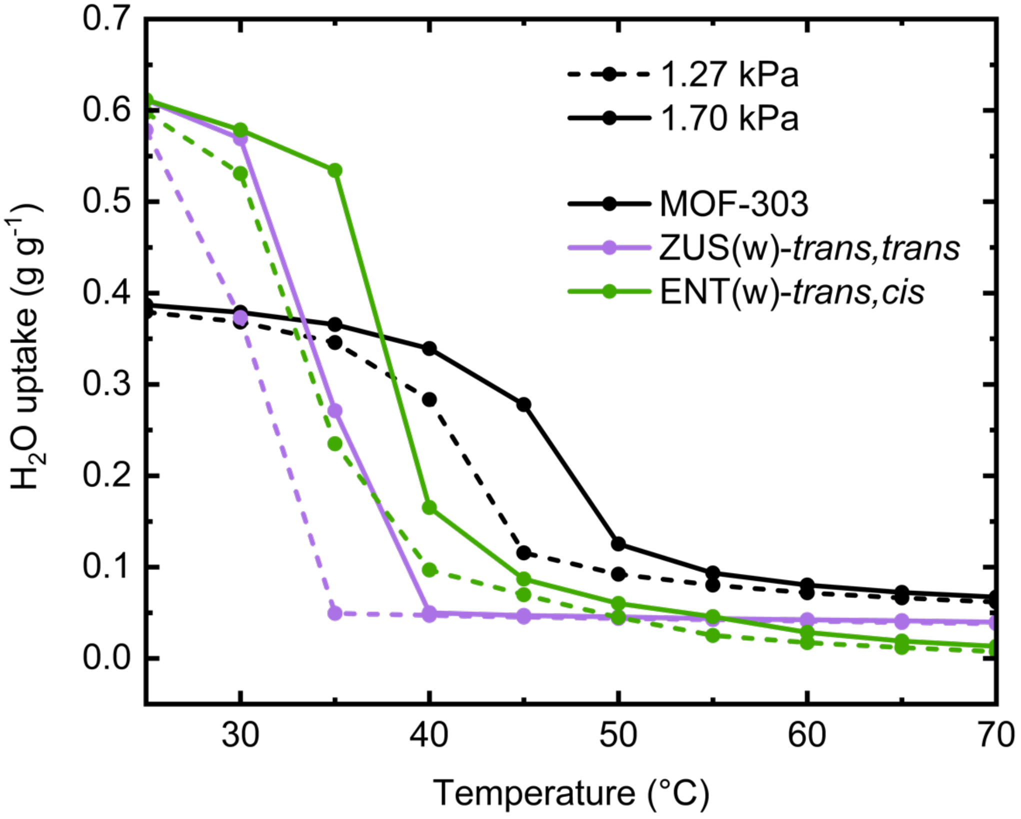

Figure S8. Simulated isobaric desorption curves for MOF-303 as well as for MOF-LA2-1 in the ZUS(w)-*trans*,*trans* and ENT(w)-*trans*,*cis* linker configurations. Data for water vapor pressures of 1.27 and 1.70 kPa are shown as dashed and solid lines, respectively.

Table S4. Tabulated data for isobaric water desorption curves calculated for MOF-303 as well as for MOF-LA2-1 in the ZUS(w)-*trans*,*trans* and ENT(w)-*trans*,*cis* linker configurations at water vapor pressures of 1.27 and 1.70 kPa determined using isobaric–isothermal GEMC simulations. The water uptake is reported in g g^{-1}, where the subscripts denote the uncertainty in the last digit(s).

Pressure (kPa)	Temperature (K)	Water uptake (g g^{-1})		
		**MOF-303**	**ZUS(w)-*trans,trans***	**ENT(w)-*trans,cis***
1.27	298	0.3794_{7}	0.5785_{23}	0.5978_{6}
1.70		0.3870_{7}	0.6114_{28}	0.6117_{7}
1.27	303	0.3683_{13}	0.3732_{92}	0.5308_{46}
1.70		0.3789_{7}	0.5691_{14}	0.5786_{29}
1.27	308	0.3459_{13}	0.0494_{1}	0.235_{12}
1.70		0.3655_{9}	0.271_{25}	0.5342_{32}
1.27	313	0.2835_{40}	0.0472_{2}	0.0968_{25}
1.70		0.3393_{14}	0.0499_{2}	0.1652_{61}
1.27	318	0.1156_{25}	0.04528_{3}	0.0696_{16}
1.70		0.2778_{50}	0.0470_{1}	0.0868_{11}
1.27	323	0.0921_{10}	0.0437_{2}	0.0448_{25}
1.70		0.1254_{24}	0.0454_{1}	0.0602_{7}
1.27	328	0.0803_{5}	0.0425_{1}	0.0252_{11}
1.70		0.0935_{19}	0.0436_{2}	0.0457_{16}
1.27	333	0.0719_{1}	0.0409_{2}	0.0172_{12}
1.70		0.0802_{3}	0.0424_{1}	0.0286_{4}
1.27	338	0.0664_{4}	0.0393_{2}	0.0120_{8}
1.70		0.0722_{2}	0.0413_{1}	0.0190_{9}
1.27	343	0.0616_{2}	0.0380_{3}	0.0076_{2}
1.70		0.0671_{3}	0.0397_{5}	0.0133_{3}

Section S4. Scanning Electron Microscopy

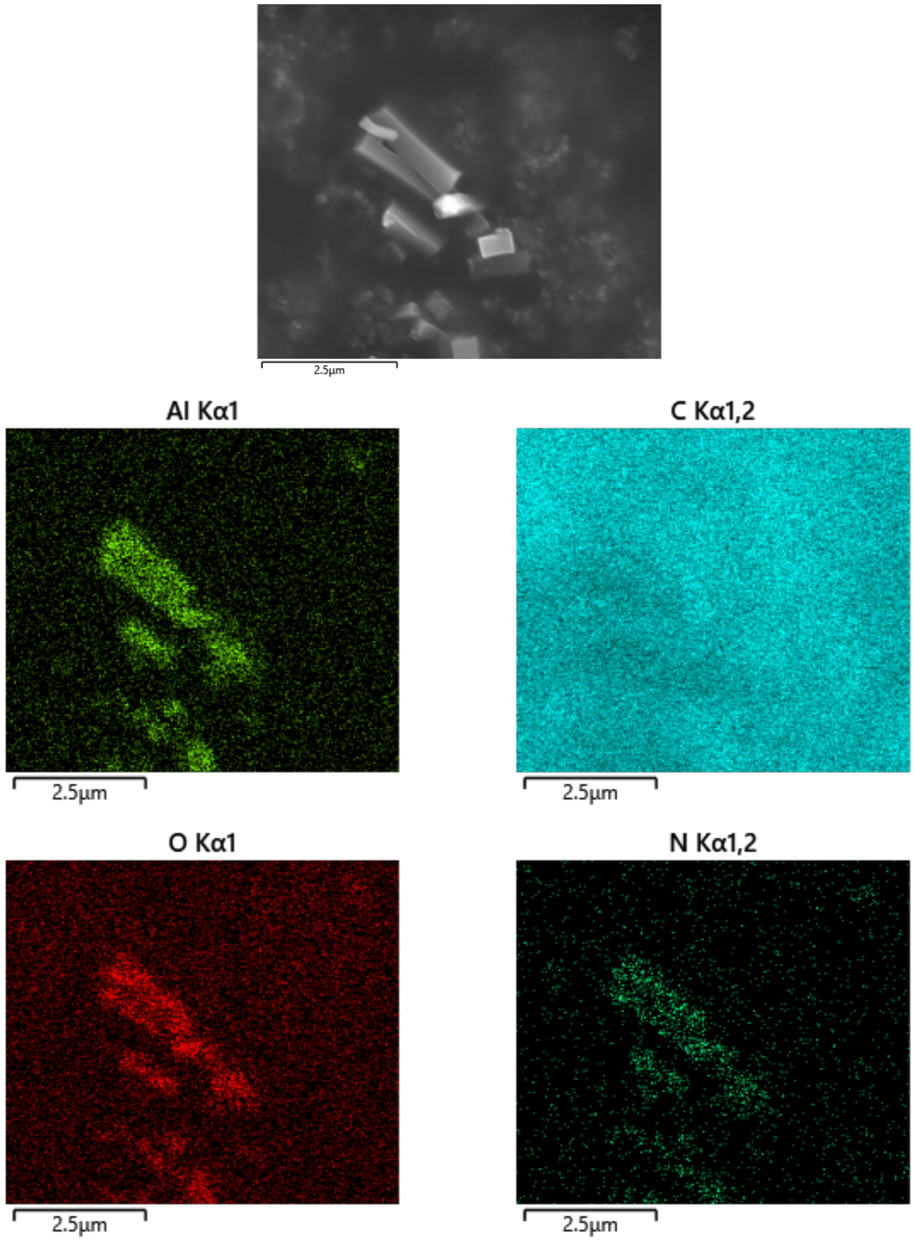

Figure S9. Scanning electron microscopy coupled with energy-dispersive X-ray spectroscopy capturing a representative fraction of the bulk material of MOF-LA2-1.

Section S5. Single-Crystal X-Ray Diffraction Analysis

Single crystals of MOF-LA2-1 were prepared in a 4-mL scintillation vial by firstly dissolving the linker H_2PZVDC (13.7 mg, 0.075 mmol, 1 equiv.) in 1.500 mL ethanol and 1.388 mL H_2O. Then, aqueous solutions of $Al_2(SO_4)_3$ (75 µL, 0.5 M, 1 equiv.) and urea (37.5 µL, 2 M, 1 equiv.) were added dropwise. The resulting clear mixture was heated in an 85 °C oven for 7 days, thus resulting in single crystals of the dimensions 10 × 10 × 30 μm^3 (Figure S10a). The respective crystals were subjected to synchrotron single-crystal X-ray diffraction (SCXRD) analysis (see Section S1 for more details). While the SCXRD data gave us insight regarding the unit cell parameters {a = 12.030(12) Å, b = 17.398(17) Å, c = 17.706(17) Å, and β = 99.33(2)°} and the SBU stereochemistry (Figure S10b), we hypothesize that due to the substantial intrinsic positional disorder of the asymmetric linker in the crystal structure, the crystallinity of this sample was relatively low, thus limiting the overall SCXRD data quality and preventing us from obtaining the exact linker configuration in MOF-LA2-1.

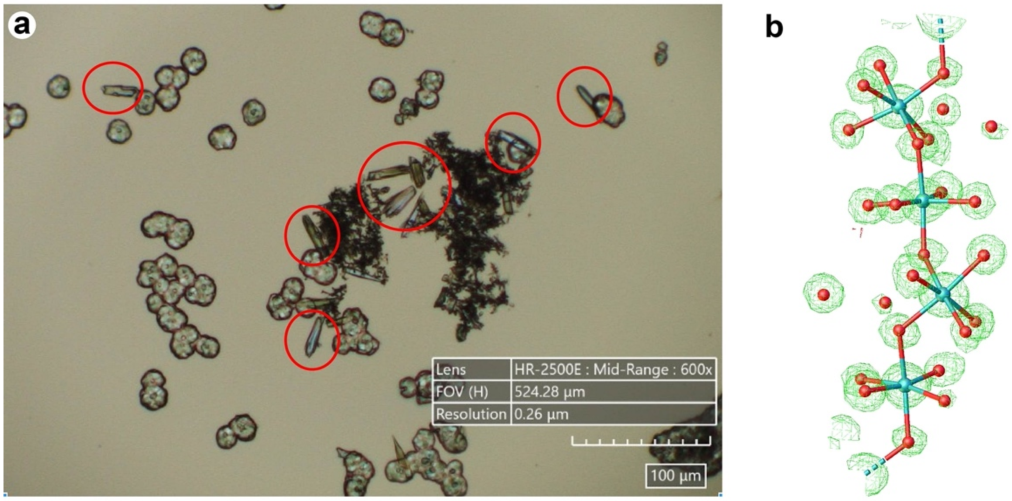

Figure S10. Single-crystal X-ray diffraction (SCXRD) analysis of MOF-LA2-1. (a) Microscope image of the sample that was subjected to SCXRD analysis. MOF-LA2-1 single crystals are circled in red. (b) Electron density map obtained from SCXRD analysis and the associated atom assignment representing an SBU consisting of alternating *cis–trans*-corner-shared AlO_6 octahedra and H_2O molecules H-bonded to it. Al, blue; O, red.

Section S6. Powder X-Ray Diffraction Analysis

MOF-LA2-1 was modeled in its most stable configuration ZUS(w)-*trans*,*trans* (see Section S3.2 for more details) in the space group $P2_1/c$ (No. 14). Using the BIOVIA Materials Studio 2020 software, Pawley refinement was conducted against the experimental powder X-ray diffraction (PXRD) data of MOF-LA2-1 to provide the unit cell parameters (a = 12.1 Å, b = 17.3 Å, c = 17.8 Å, β = 98.6°) with good agreement factors (R_p = 6.01%, R_{wp} = 3.47%).

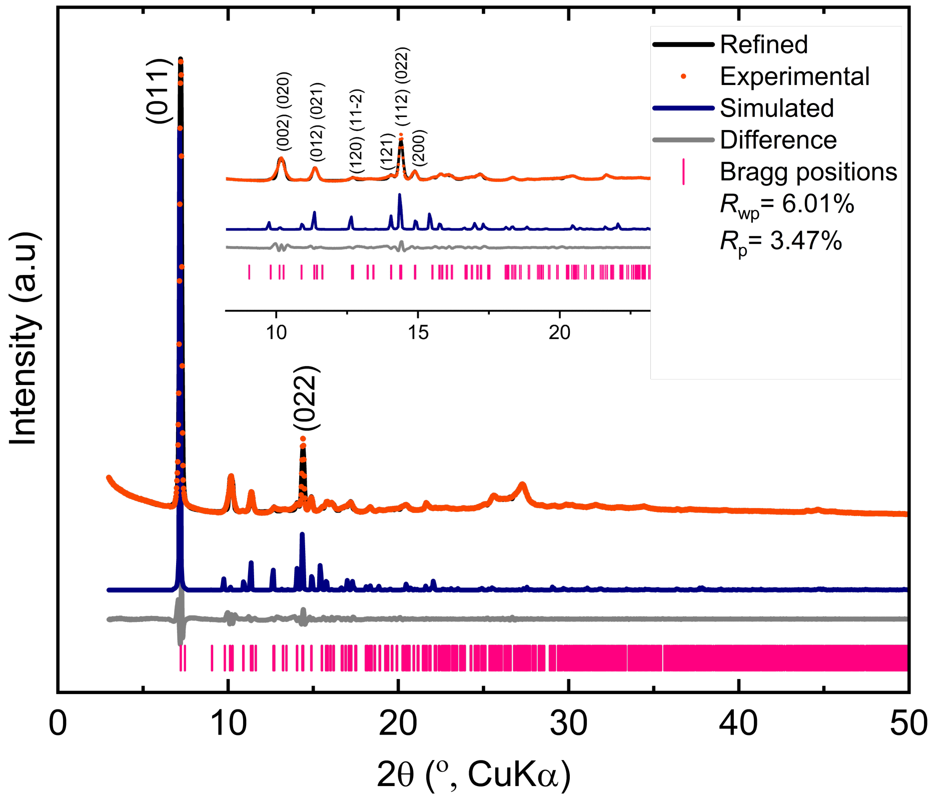

Figure S11. Pawley refinement of MOF-LA2-1 in the ZUS(w)-*trans*,*trans* configuration against the experimental PXRD pattern of MOF-LA2-1 prepared via solvothermal synthesis. Major peaks are indexed.

Table S5. Fractional atomic coordinates of MOF-LA2-1 in the ZUS(w)-*trans*,*trans* configuration {monoclinic, $P2_1/c$ (No. 14), a = 12.1 Å, b = 17.3 Å, c = 17.8 Å, β = 98.6°}, obtained through Pawley refinement of the respective structural model against the experimental PXRD data.

Atom	x	y	z
Al1	0.76311	0.44853	0.56349
O1	0.87602	0.37148	0.59894
O5	0.32121	0.90698	0.86343
O9	0.84561	0.48586	0.48898
O13	0.16017	0.0168	0.8619
O17	0.65181	0.52177	0.5335
O21	0.0299	0.39811	0.54322
O25	0.29999	0.62728	0.5097
O29	0.99308	0.04209	0.90028
O33	0.46315	0.57564	0.57123
O37	0.49995	0.92372	0.9252
H1	0.81897	0.46382	0.43912
H5	0.67785	0.5628	0.50385
H9	0.66161	0.72965	0.79353
H13	0.20018	0.31345	0.5993
H17	0.91531	0.2319	0.68518
H21	0.32241	0.80742	0.73575
H25	0.04688	0.86728	0.21109
H29	0.80258	0.8949	0.23745
H33	0.54787	0.8485	0.1778
H37	0.31663	0.77609	0.11714
N1	0.58429	0.75718	0.78974
N5	0.57668	0.81099	0.8419
N9	0.18626	0.22128	0.66862
N13	0.14658	0.28087	0.625
C1	-2.2E-4	0.23807	0.67223
C5	0.97444	0.3595	0.58539
C9	0.38451	0.62499	0.56383
C13	0.06598	0.05092	0.85582
C17	0.42659	0.89376	0.87431
C21	0.40959	0.79866	0.76114
C25	0.09705	0.19393	0.69839
C29	0.03478	0.2937	0.62543
C33	0.46899	0.83654	0.82503
C37	0.48692	0.7471	0.73872
C41	0.88589	0.8682	0.2474
C45	0.96303	0.89247	0.20561
C49	0.47802	0.8084	0.1784
C53	0.38857	0.81489	0.12221
Al5	0	0.5	0.5
Al7	0.5	0.5	0.5

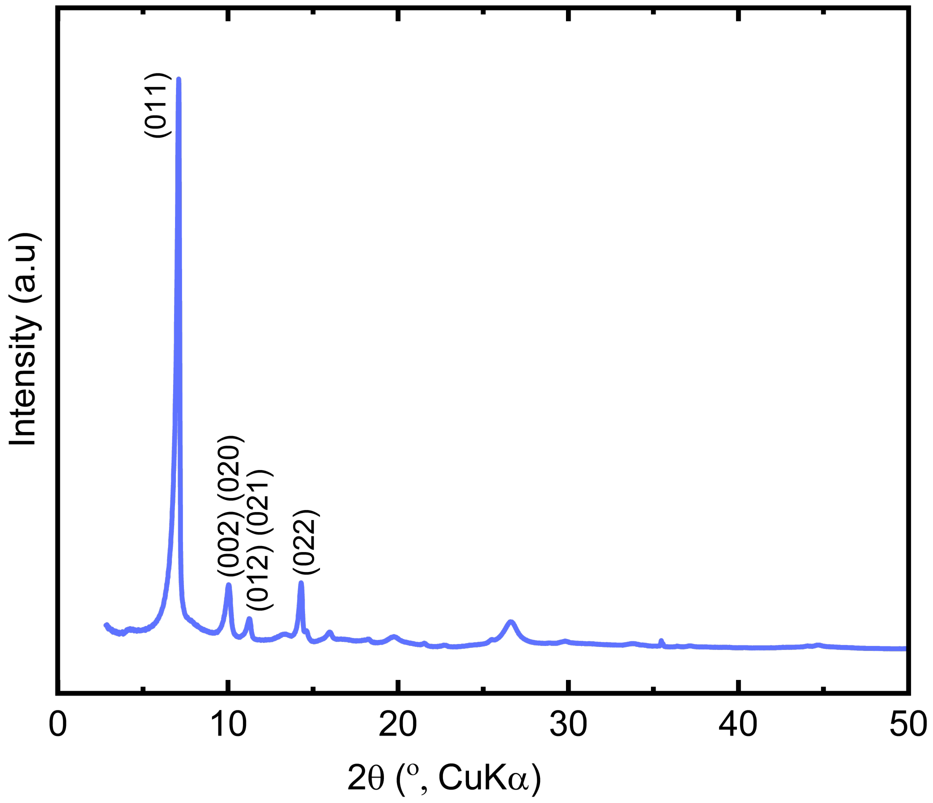

Figure S12. Powder X-ray diffraction pattern of MOF-LA2-1 prepared by using the reflux-based green synthesis method.

Section S7. Thermogravimetric Analysis

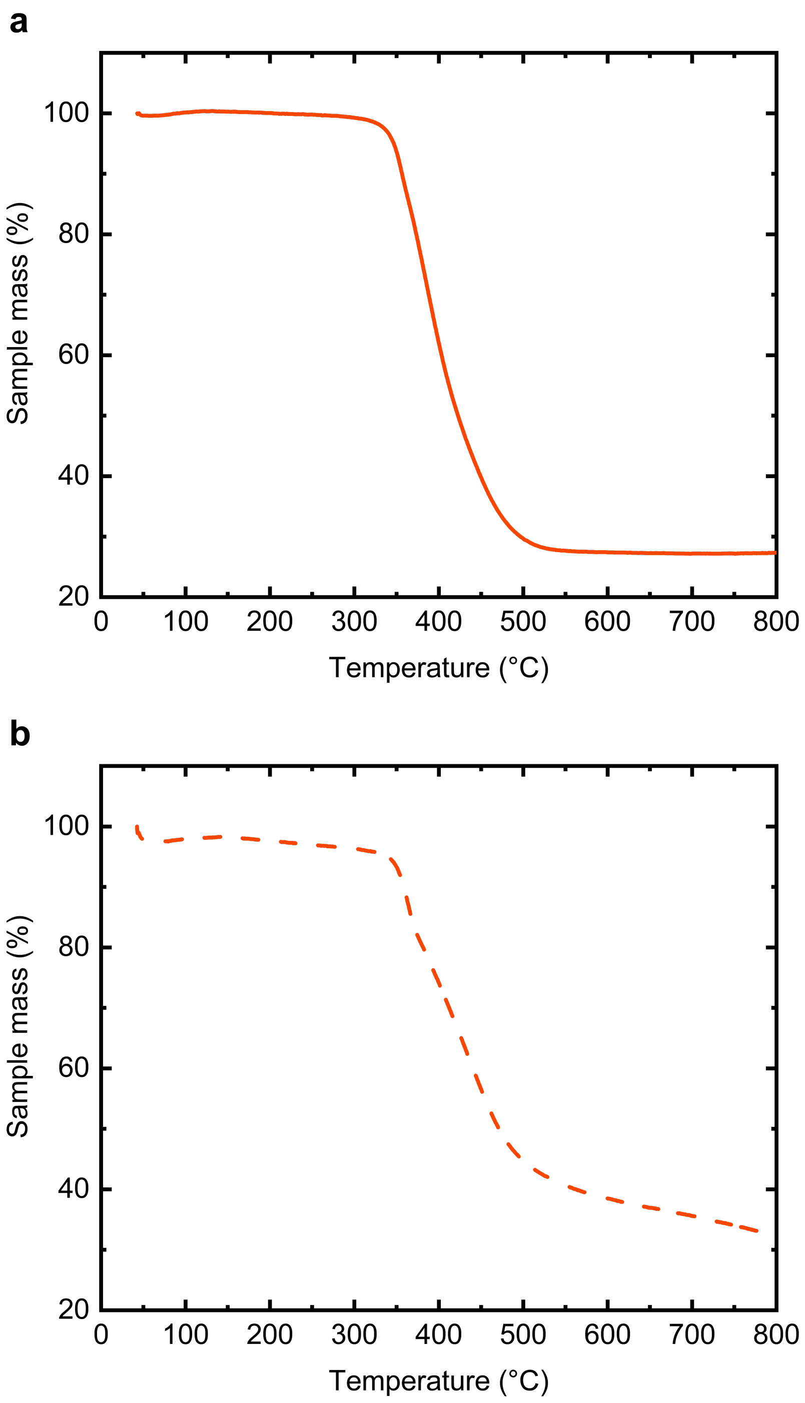

Figure S13. Thermogravimetric analyses of MOF-LA2-1 conducted under air (a) and argon atmosphere (b).

Section S8. Nitrogen Sorption Analysis

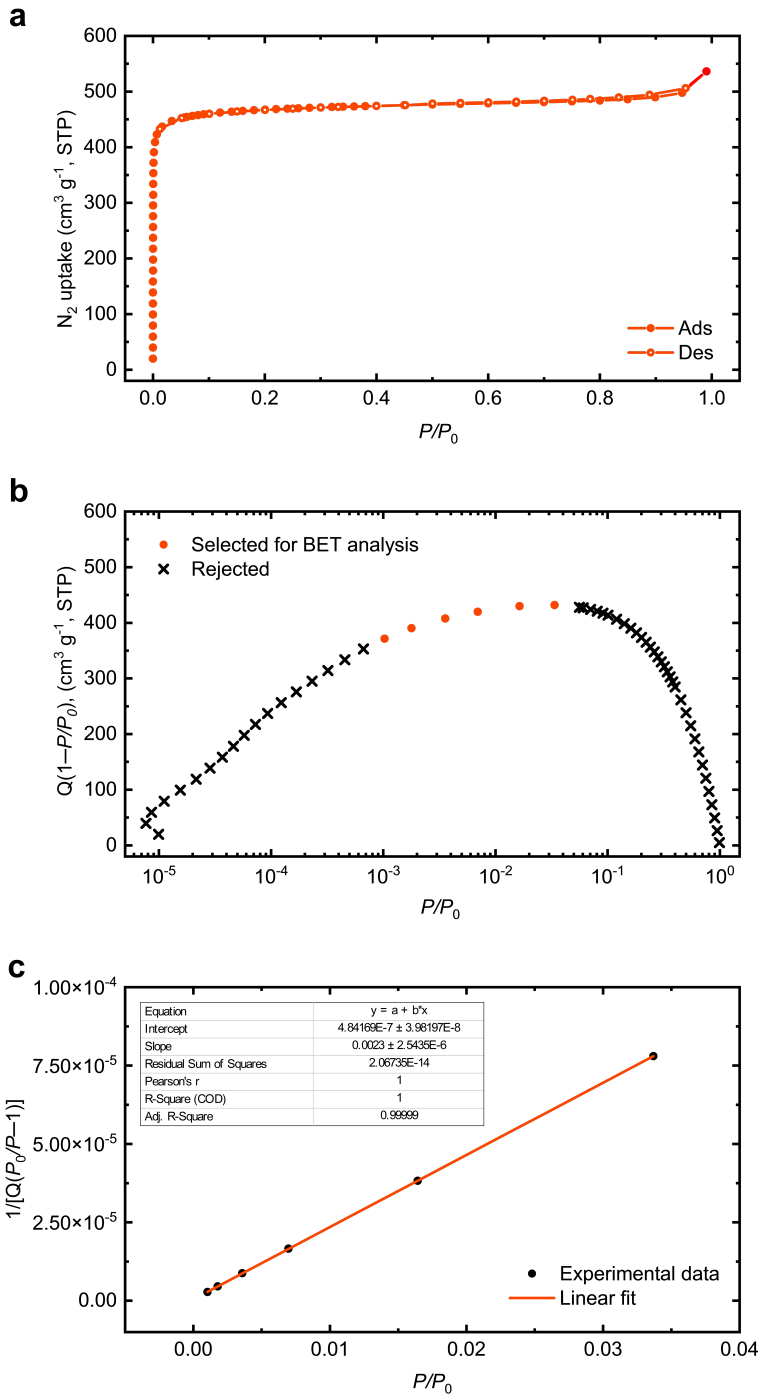

Figure S14. Nitrogen sorption analysis of MOF-LA2-1 prepared via solvothermal synthesis. (a) N_2 sorption isotherm at 77 K. (b) Rouquerol plot for determination of the appropriate pressure range for BET analysis. (c) BET plot yielding a BET surface area of 1892 ± 2 m^2 g^{-1}. P, nitrogen pressure; P_0 = 1 atm; STP, standard temperature and pressure.

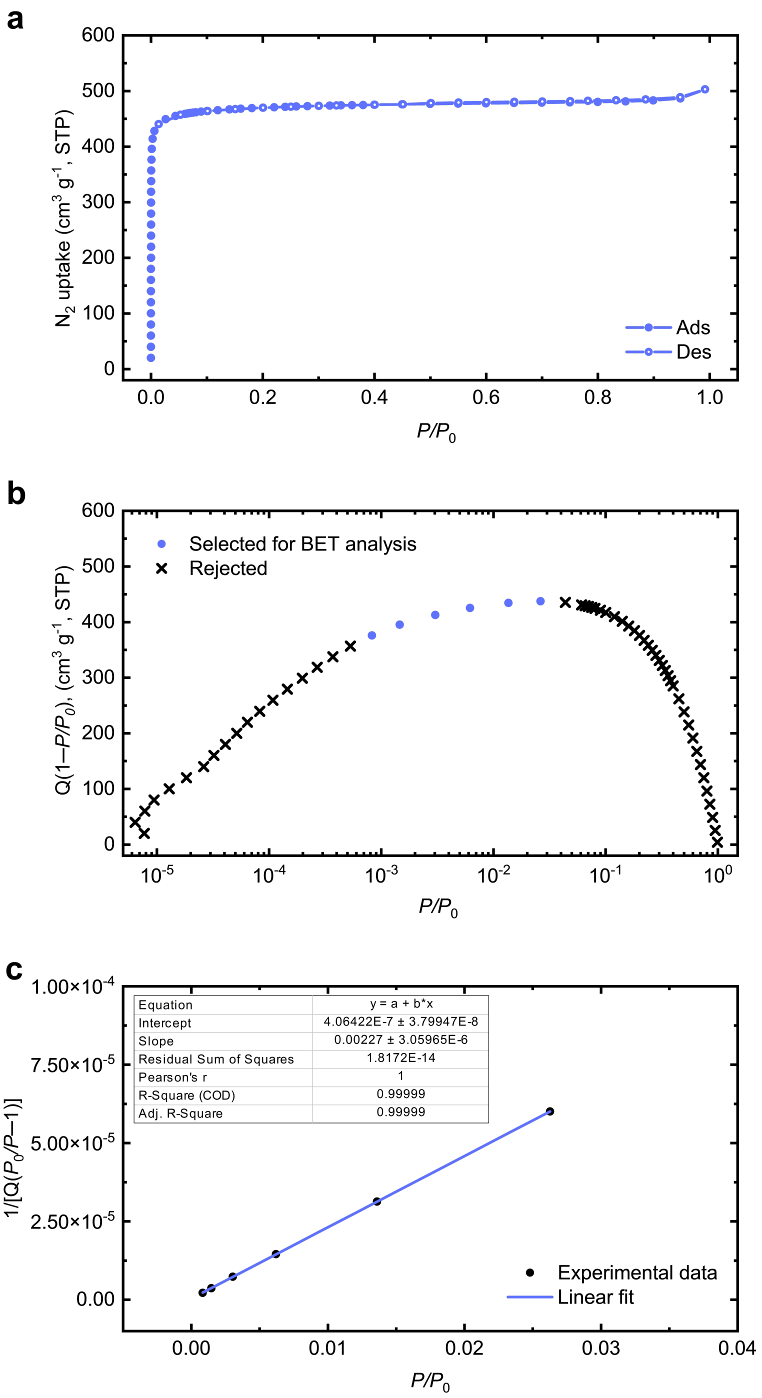

Figure S15. Nitrogen sorption analysis of MOF-LA2-1 prepared via reflux-based green synthesis: (a) N_2 sorption isotherm at 77 K. (b) Rouquerol plot for determination of the appropriate pressure range for BET analysis. (c) BET plot yielding a BET surface area of 1916 ± 2 m^2 g^{-1}. *P*, nitrogen pressure; P_0 = 1 atm; STP, standard temperature and pressure.

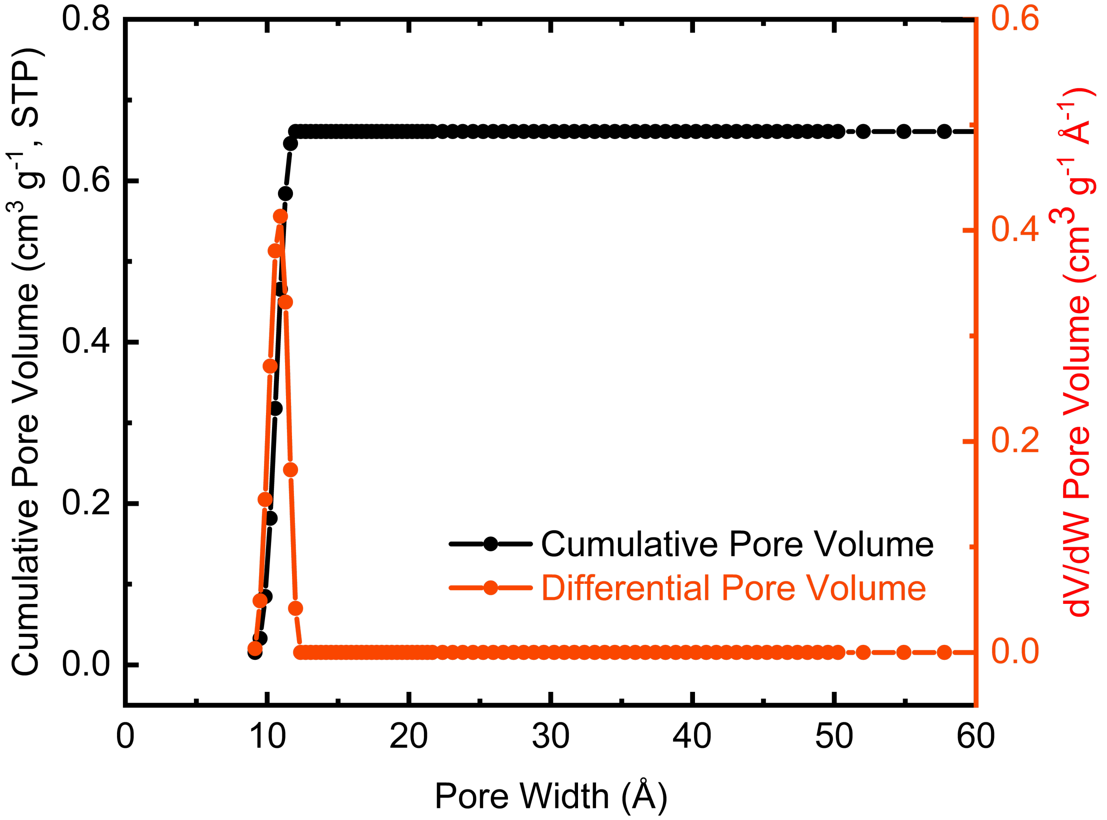

Figure S16. Pore size distribution of MOF-LA2-1 derived from the N_2 sorption isotherm measured at 77 K. Applying classical density functional theory with a kernel for oxide surfaces with cylindrical pores yields a pore width of 11 Å and a cumulative pore volume of 0.67 cm^3 g^{-1}.

Section S9. Water Sorption Analysis

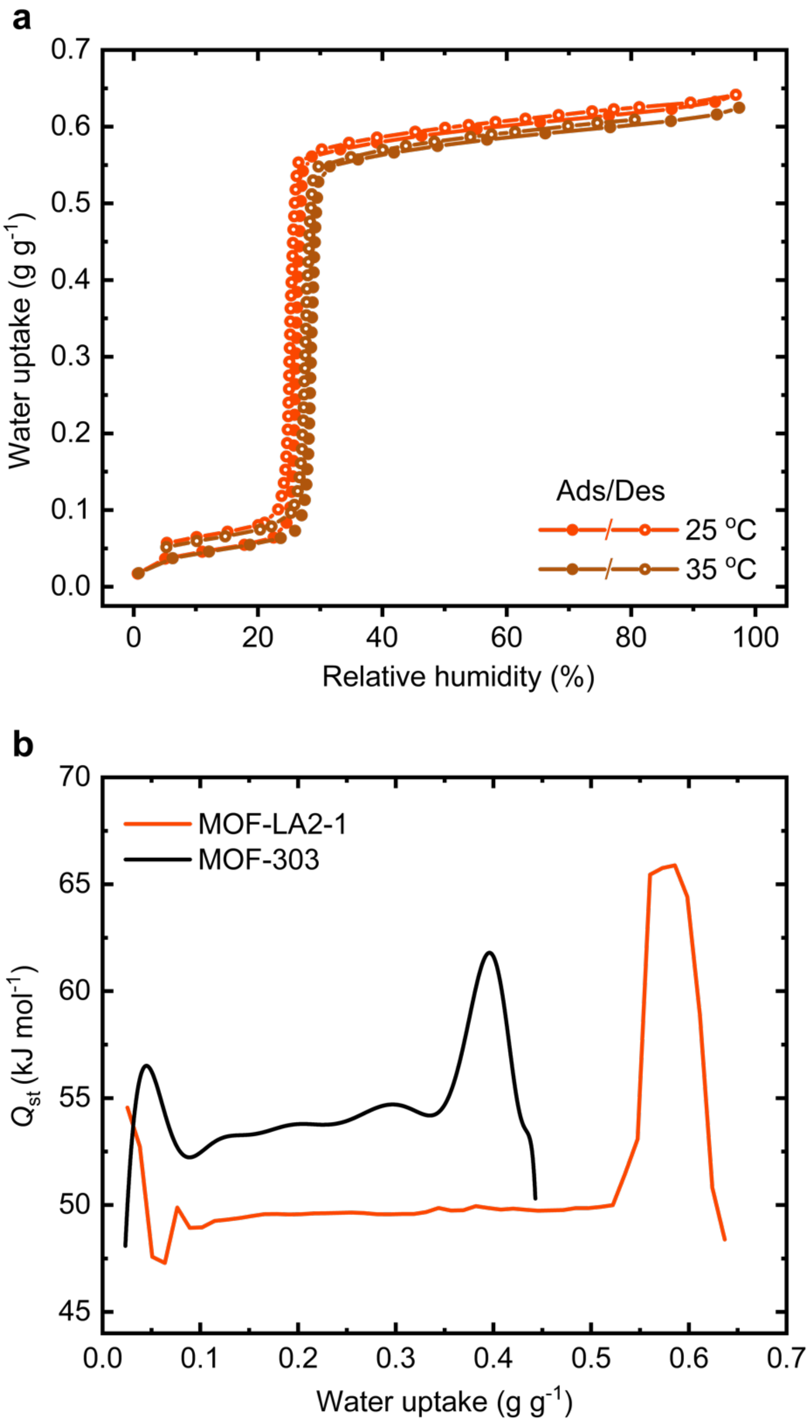

Figure S17. Water sorption analysis of MOF-LA2-1 prepared via solvothermal synthesis. (a) Water sorption isotherms of MOF-LA2-1 at 25 and 35 °C. (b) Heat of adsorption Q_{st} in dependence of water loading estimated with the Clausius–Clapeyron equation.

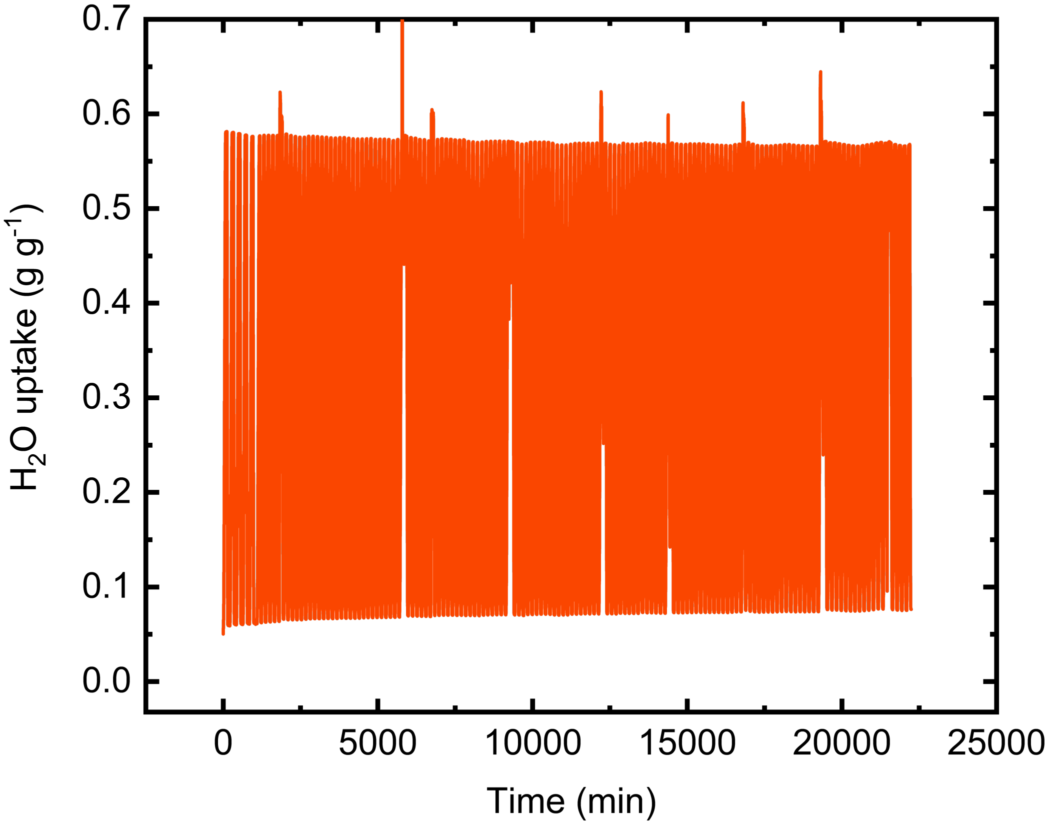

Figure S18. 150 water vapor adsorption–desorption cycles conducted on MOF-LA2-1 at a water vapor pressure of 1.70 kPa and initiated through temperature swing adsorption between 30 and 45 °C. The time per cycle was ~150 min.

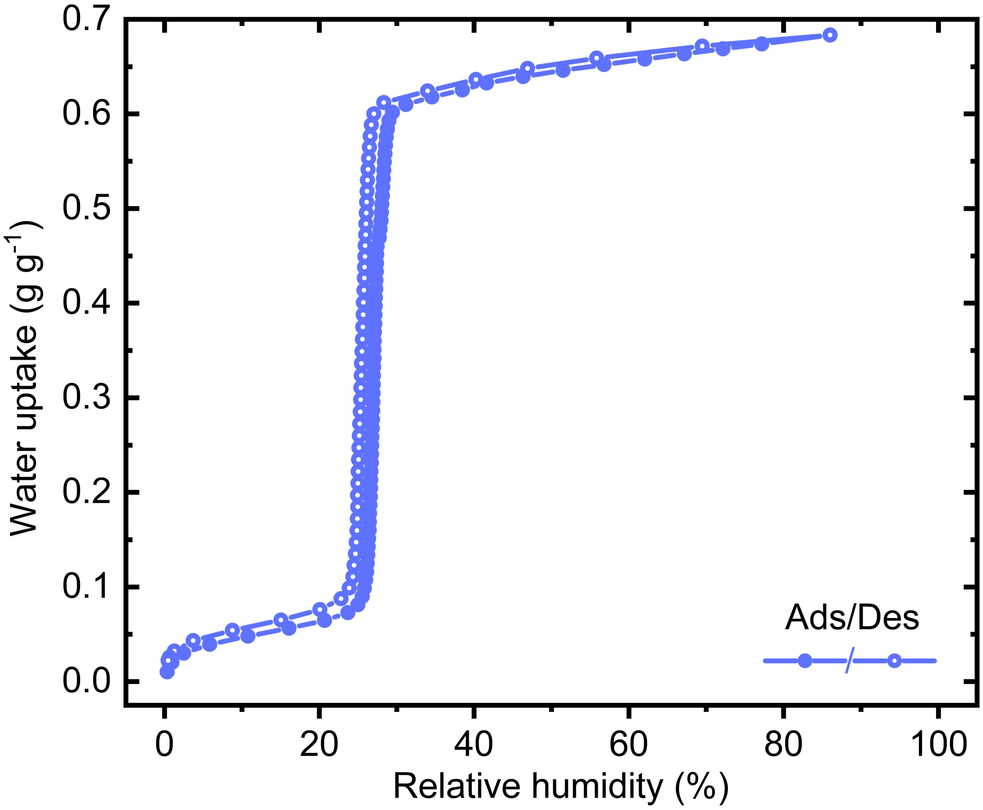

Figure S19. Water sorption analysis of MOF-LA2-1 at 25 °C prepared by using the reflux-based green synthesis method.

Section S10. References

(1) Hanikel, N.; Pei, X.; Chheda, S.; Lyu, H.; Jeong, W.; Sauer, J.; Gagliardi, L.; Yaghi, O. M. Evolution of Water Structures in Metal-Organic Frameworks for Improved Atmospheric Water Harvesting. *Science* **2021**, *374* (6566), 454–459.

(2) Zheng, Z.; Hanikel, N.; Lyu, H.; Yaghi, O. M. Broadly Tunable Atmospheric Water Harvesting in Multivariate Metal–Organic Frameworks. *J. Am. Chem. Soc.* **2022**, *144* (49), 22669–22675.

(3) Bruker AXS Inc. APEX3. Madison, WI 2018.

(4) Bruker AXS Inc. SAINT. Madison, WI 2018.

(5) Bruker AXS Inc. SADABS. Madison, WI 2016.

(6) Sheldrick, G. M. SHELXT – Integrated Space-Group and Crystal-Structure Determination. *Acta Crystallogr. Sect. A Found. Adv.* **2015**, *71* (1), 3–8.

(7) Sheldrick, G. M. Crystal Structure Refinement with SHELXL. *Acta Crystallogr. Sect. C Struct. Chem.* **2015**, *71* (1), 3–8.

(8) Dolomanov, O. V.; Bourhis, L. J.; Gildea, R. J.; Howard, J. A. K.; Puschmann, H. OLEX2 : A Complete Structure Solution, Refinement and Analysis Program. *J. Appl. Crystallogr.* **2009**, *42* (2), 339–341.

(9) Kresse, G.; Furthmüller, J. Efficiency of Ab-Initio Total Energy Calculations for Metals and Semiconductors Using a Plane-Wave Basis Set. *Comput. Mater. Sci.* **1996**, *6* (1), 15–50.

(10) Perdew, J. P.; Burke, K.; Ernzerhof, M. Generalized Gradient Approximation Made Simple. *Phys. Rev. Lett.* **1996**, *77* (18), 3865–3868.

(11) Grimme, S.; Antony, J.; Ehrlich, S.; Krieg, H. A Consistent and Accurate Ab Initio Parametrization of Density Functional Dispersion Correction (DFT-D) for the 94 Elements H-Pu. *J. Chem. Phys.* **2010**, *132* (15), 154104.

(12) Panagiotopoulos, A. Z. Direct Determination of Phase Coexistence Properties of Fluids by Monte Carlo Simulation in a New Ensemble. *Mol. Phys.* **1987**, *61* (4), 813–826.

(13) Panagiotopoulos, A. Z.; Quirke, N.; Stapleton, M.; Tildesley, D. J. Phase Equilibria by Simulation in the Gibbs Ensemble. *Mol. Phys.* **1988**, *63* (4), 527–545.

(14) Siepmann, J. I.; Martin, M. G.; Chen, B.; Wick, C.; Stubbs, J. M.; Potoff, J. J.; Eggimann, B. L.; McGrath, M. J.; Zhao, X. S.; Anderson, K. E.; Rafferty, J. L.; Rai, N.; Maerzke, K.

A.; Keasler, S. J.; Bai, P.; Fetisov, E. O.; Shah, M. S.; Chen, Q. P.; DeJaco, R. F.; Chen, J. L.; Xue, B.; Bunner, C.; Sun, Y. Z. S.; Josephson, T. R. Monte Carlo for Complex Chemical Systems–Minnesota. University of Minnesota: Minneapolis, MN 2020.

(15) Chheda, S.; Jeong, W.; Hanikel, N.; Gagliardi, L.; Siepmann, J. I. Monte Carlo Simulations of Water Adsorption in Aluminum Oxide Rod-Based Metal–Organic Frameworks. *in revision at J. Phys. Chem. C.*

(16) Jorgensen, W. L.; Chandrasekhar, J.; Madura, J. D.; Impey, R. W.; Klein, M. L. Comparison of Simple Potential Functions for Simulating Liquid Water. *J. Chem. Phys.* **1983**, *79* (2), 926–935.

(17) Maerzke, K. A.; Schultz, N. E.; Ross, R. B.; Siepmann, J. I. TraPPE-UA Force Field for Acrylates and Monte Carlo Simulations for Their Mixtures with Alkanes and Alcohols. *J. Phys. Chem. B* **2009**, *113* (18), 6415–6425.
